# Approaches to inducing mental fatigue: A systematic review and meta-analysis of (neuro)physiologic indices

**DOI:** 10.3758/s13428-025-02620-7

**Published:** 2025-02-26

**Authors:** Stephen P. J. Goodman, Blake Collins, Kathleen Shorter, Ashleigh T. Moreland, Christopher Papic, Adam S. Hamlin, Brendon Kassman, Frank E. Marino

**Affiliations:** 1 School of Science and Technology, University of New England, Armidale, New South Wales Australia; 2 Holsworth Research Initiative, La Trobe Rural Health School, La Trobe University, Bendigo, Australia; 3 School of Health Sciences, Swinburne University of Technology, Melbourne, Victoria Australia; 4 Re-MIND Institute, Sunbury, Victoria Australia; 5 RECOVER Injury Research Centre, Faculty of Health and Behavioural Sciences, The University of Queensland, Brisbane, Queensland Australia; 6 School of Rural Medicine, Charles Sturt University, Orange, New South Wales Australia

**Keywords:** Mental fatigue, Physiology, Neurophysiology, Methodology

## Abstract

Mental fatigue is a transient psychophysiological state characterized by impaired cognition and behavior across a range of dynamic contexts. Despite increasing interest in this phenomenon, its (neuro)physiologic representations remain unclear. This systematic review aimed to quantify the range of (neuro)physiologic outcomes and methodologies used to investigate mental fatigue in laboratory-based settings. Across the 72 studies meeting our inclusion criteria, we identified 30 unique physiologic, four visual outcomes, and the application of several neuroimaging techniques investigating neuronal function. Mental fatigue increased heart rate, systolic and diastolic blood pressure, mean arterial pressure, low frequency, and root mean square of successive differences (RMSSD), and reduced standard deviation of normal-to-normal intervals (SDNN) (all *P* ≤ 0.04) when compared with controls. Applying electroencephalography to investigate delta, theta, and alpha bandwidths may provide useful insights into this phenomenon, and functional near-infra-red spectroscopy to right-lateralized frontoparietal regions would be helpful to investigate cortical activity change in response to mental fatigue. More data are needed across a range of methodological contexts in order to further determine the (neuro)physiological manifestations of mental fatigue. However, this review provides direction to researchers and will assist them in navigating and considering the range of options available.

**Supplementary Information:**

The online version contains supplementary material available at 10.3758/s13428-025-02620-7.

## Introduction

Mental fatigue is an experience encountered by many and experientially characterized by lethargy, tiredness, and an aversion to continued task engagement (Van Cutsem et al., 2017). However, a scientific definition for this phenomenon remains elusive due to varied inter-related constructs and manifestations, creating challenges for its investigation (Hassan et al., 2024). Such constructs include self-regulation, ego-depletion, and cognitive or mental fatigue. Recently, MacMahon et al. (2023) delineated these, highlighting that self-regulation or control may reflect a limited capacity to adapt one’s behavior and overcome habitual responses to attain a desired goal (Baumeister et al., 2007). Ego depletion refers to a temporarily diminished capability to engage in a volitional act following an initial effortful activity (Baumeister et al., 1998). While mental or cognitive fatigue is regarded as a transient psychophysiological state of amotivation, lethargy, and diminished performance capabilities due to sustained cognitive effort (Van Cutsem et al., 2017). Overlaps in these constructs have led to varied perspectives on whether these reflect common or separate phenomena (Brown et al., 2019; Forestier & Chalabaev, 2020; Habay et al., 2023), and has likely stalled our understanding of this collective phenomenon. For the purposes of this review, we use the term ‘mental fatigue’ to encompass the spectrum of related literature concerning this, cognitive fatigue, ego-depletion, and self-control/ regulation.

Mental fatigue is thought to arise during instances of sustained or compressed cognitive workload (Borragan et al., 2017), and may generally be experienced by individuals in a range of contexts. This phenomenon transcends repetitive computer-based activities, for instance, occupations involving transportation or surgery may result in extensive time-on-task with a potentially high cost placed on decisions and may result in mental fatigue (Kunasegaran et al., 2023; van der Linden, 2011). Within sporting contexts, increased cognitive load may also occur throughout a match due to rapid and repetitive decision-making under high-pressure situations (Coutts, 2016). Further, mental demand may also accrue chronically in athletes due to the array of cognitively stimulating activities they perform in addition to acute game-specific load, e.g., interviews, training activities, and sponsorship requirements (Russell et al., 2019a, 2019b; Russell et al., 2019a, 2019b). A mentally fatigued state is also likely to influence an array of performance contexts. For instance, endurance capacity has been shown to decline (Brown et al., 2019; Giboin & Wolff, 2019; Van Cutsem et al., 2017), number of repetitions and isometric resistance performance impaired (Alix-Fages et al., 2022; Brown et al., 2019), and sustained and sequential cognitive performance may also diminish (Habay, Van Cutsem, et al., 2021b; Oliver et al., 2025; van der Linden et al., 2003). Knowing that mental fatigue is prevalent in a range of contexts and may have a profound influence in several performance domains, a need to understand this phenomenon continues to grow.

So how is mental fatigue elicited and what should researchers monitor in order to determine the presence of this psychophysiological state? The answer to the first part of this question is straight forward and a common approach exists within laboratory-based settings, whereby participants undertake cognitively demanding work for an arbitrary period of time. However, heterogeneity exists in the type of task(s) that could be used to induce mental fatigue. Popular choices within the literature include variants of the Stroop task (Goodman & Marino, 2021; Smith et al., 2019; Van Cutsem et al., 2019), AX-continuous performance task (AX-CPT) (Marcora et al., 2009; Smith et al., 2019), N-back tests (Dallaway et al., 2022; Tanaka et al., 2012), psychomotor vigilance task (PVT) (Kowalski et al., 2022; Smith et al., 2019), or a combination of tasks (Clark et al., 2019; Hassan et al., 2023). These serve as the intervention and outcomes are generally weighed against control tasks incorporating a cognitively stimulating activity (e.g., video, simpler cognitive task, reading etc.) to discern the emergence of mental fatigue. Further uncertainty exists concerning the duration of interventions; though selecting an arbitrary duration likely assumes participants will respond equally to the stressor. Some have suggested interventions should be a minimum of 30 min (Pageaux & Lepers, 2018; Van Cutsem et al., 2017). However, as described by Brown et al. (2019), this recommendation overlooks another related body of research adopting shorter-duration tasks; ego-depletion. Interestingly, one analysis (Brown et al., 2019) found mental fatigue to have a debilitating effect on sequential physical performance irrespective of whether studies were grouped by < 30 or ≥ 30 min, when incorporating relevant ego-depletion literature. This is supported by other studies (Borragan et al., 2017; Gantois et al., 2020; O'Keeffe et al., 2020), which also incorporated tasks using durations shorter than 30 min (Van Cutsem et al., 2017).

With respect to how mental fatigue manifests, and whether or not it can it be measured, such fatigue is thought to have both subjective and objective manifestations (Boksem & Tops, 2008). Subjectively, individuals may experience increased lethargy, reduced attentiveness, lack of energy, or perceive themselves to apply greater effort to a task (Van Cutsem et al., 2017). Self-report measures will often be obtained prior to, during, and following an intervention and comparisons between conditions or groups will then be made to determine whether participants experienced mental fatigue. Such insights are undoubtedly valuable contributions to the experience of fatigue, but there is the potential for subjective bias (Boksem & Tops, 2008). Studies often accept this limitation and opt to compliment self-report measures with objective indices. Objectively, mental fatigue is thought to be recognized through fluctuations in cognitive or behavioral task performance (e.g., slowed response time, reduced accuracy, altered movement economy etc.), or via (neuro)physiologic alterations (Kunasegaran et al., 2023; Van Cutsem et al., 2017). The former is of considerable importance, as these indices are likely those related to performance output settings (e.g., work productivity, cognitive or motor performance etc.). However, these may act as the net product of altered neurophysiology (Wang et al., 2016) or shifts in motivation or applied effort (Boksem & Tops, 2008; Herlambang et al., 2019). Comparatively, (neuro)physiologic representations of such mental fatigue (and the focus of this review) may be more resistant to such conflation and bias, and are key to understanding its biological basis. At a glance, several biomarkers have been used within the literature. For instance, classic measures such as heart rate (HR), or changes in biochemistry (e.g., glucose, lactate, or cortisol) are common (Clark et al., 2019; Marcora et al., 2009; Schücker & MacMahon, 2016). Similarly, features of heart rate variability are also reported, including low-frequency power, the standard deviation of normal-to-normal intervals (SDNN), or changes between R-R intervals (Head et al., 2017; Smith et al., 2019). Given the probable neurological basis of mental fatigue, investigations into neuronal function using technologies such as electroencephalography (EEG), functional near-infrared spectroscopy (fNIRS), and functional magnetic resonance imaging (fMRI) are also popular choices to examine electrical or hemodynamic changes and infer regional activity fluctuations in response to mental fatigue (Goodman & Marino, 2021; Smith et al., 2019; Van Cutsem et al., 2022). Furthermore, monitoring eye metrics is becoming more prevalent as this technology becomes increasingly refined and user (Bafna & Hansen, 2021).

With the range of (neuro)physiological outcomes available to the researcher to explore the effects of mental fatigue, it can be confronting and difficult to determine which might be worthwhile integrating. Recently, a comparative analysis of several assessments and tools for the early detection of mental fatigue has been conducted (Kunasegaran et al., 2023). In addition to subjective and behavioral elements, several of the outcomes outlined above are also discussed (e.g., EEG, heart rate variability, cortisol, and saccadic eye movements). Although the intention of their review was to provide insight into prospective biological markers of use for monitoring mental fatigue, the selective focus on these ignores the prospect of other potential (neuro)physiologic manifestations. Additionally, there may be benefits to researchers in understanding what outcomes and measures may not be influenced by this phenomenon, as this may enable greater resource and time efficiency. Thus, the primary aim of this systematic review and meta-analysis was to examine the breadth of (neuro)physiologic indices that have been used to investigate mental fatigue and identify which may be (in)effective in determining its manifestation. Additionally, we sought to examine the range of cognitive tasks and control condition types used in mental fatigue interventions; as they relate to studies examining (neuro)physiological outcomes.

## Methods

### Study protocol

A protocol document was developed a priori using the 2020 Preferred Reporting Items for Systematic Reviews and Meta-Analysis (PRISMA) (Page et al., 2021), and registered on PROSPERO (CRD42021288158). Due to the volume of literature uncovered, we opted to stage the research aims of this project and examine physiologic, behavioral, subjective, and sequential tasks as separate but related companion papers. This review examines (neuro)physiologic indices of mental fatigue.

### Eligibility criteria

For inclusion, studies were required to: (1) Include an apparently healthy population; (2) Incorporate a randomized controlled design; (3) Use a mental fatigue intervention including a cognitive task (e.g., Stroop task or PVT); (4) Include a control intervention involving a ‘cognitively stimulating’ activity (e.g., media viewing or undertaking a less demanding cognitive task); (5) Report objective or subjective measures of mental fatigue (e.g., cognitive performance, cortical hemodynamic function, self-reported exhaustion etc.); (6) Include no confounding variables during the mental fatigue and/or control interventions (e.g., sleep deprivation, hypoxia, or interleaved designs); (7) Be available as a full text in English.

### Systematic search strategy

**Fig. 1:**
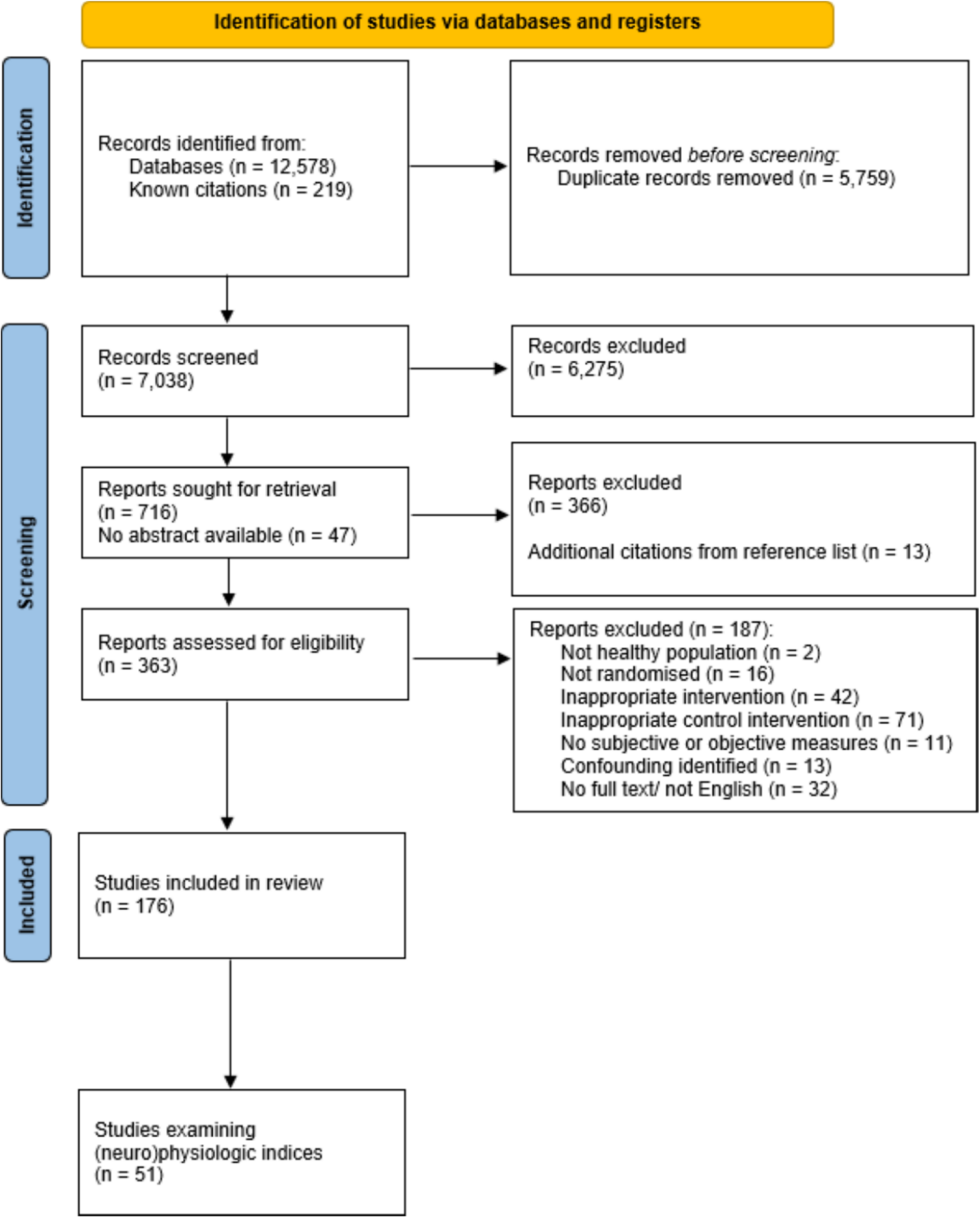
Preferred Reporting Items for Systematic Reviews and Meta-Analysis (PRISMA) flow diagram outlining the implemented search strategy and eligibility process (Page et al., 2021). The follow-up literature search is provided in Supplementary Fig. 1 (https://osf.io/97xad/)

Figure 1 reports the initial search strategy and eligibility process. In November 2021, two authors completed the search strategy (“mental fatigue” OR “cognitive fatigue” OR “cognitive exertion” OR “mental exertion” OR “mental strain” OR “cognitive strain” OR “ego depletion”) via Embase, PubMed, the Web of Science, and SportsDiscus databases, and captured the first 200 citations from Google Scholar (Bramer et al., 2017). Searches were limited to human participants, written in English, and no date restrictions were used. 12,578 studies were captured and imported into reference management software (EndNote v20, Clarivate, USA), alongside 219 studies known to the authors. Following the removal of duplicates, the title of 7038 citations were independently screened, followed by 716 abstracts, and finally 363 full-text articles were reviewed against the eligibility criteria. Reference lists of full-text studies were examined for literature not captured (*n* = 13 additional citations), which the full-text were also examined. Included in the review were 176 citations. An independent third reviewer settled any conflicts throughout the literature search (*n* = 24; 7% conflict of full texts).

From the initial search, a subset of literature examining (neuro)physiological indices of mental fatigue was partitioned (*n* = 51 studies; Fig. 1). Two follow-up literature searches were conducted. The first was to capture literature published since the initial search (Supplementary Fig. 1; https://osf.io/97xad/). Parameters for this search were identical to those above, but with the restriction of being published between November, 2021, to current (February, 2023) and criteria 5 was altered to: “a (neuro)physiological outcome measure of mental fatigue was examined during the interventions”. From a further 2168 citations after the removal of duplicates. Two authors reviewed the titles and abstracts of these citations and examined 159 citations for inclusion. An additional ten citations were found to meet the inclusion criteria, bringing the total number of included studies examining (neuro)physiological outcomes to 61 studies. Based on feedback from an anonymous reviewer a third literature search was conducted. Here we combined two search strategies before undertaking literature screening. The first included a separate strategy (“cognitive effort” OR “mental effort”) through the above-mentioned databases. No date restrictions were used, but literature was limited to human participants. These outputs were combined with a further search we completed to distinguish citations published since the second literature search (February 2023 to June, 2024). Combining the searches in citation management software (Covidence, Veritas Health Innovation, Australia), two authors examined a further 4659 titles and abstracts, and 227 full texts (Supplementary Fig. 2; https://osf.io/97xad/). To the 61 articles already included, a further 11 were added; making a total of 72 studies reporting (neuro)physiologic outcomes.

### Risk of bias

The revised Cochrane risk of bias 2 for independent samples or the risk of bias 2 for crossover trials was applied as appropriate to all eligible studies. Assessment was made at the outcome level independently by two authors, and conflicts resolved by a third. All procedures were in accordance with published norms (Sterne et al., 2019).

### Data extraction

Data extraction was performed by one author and reviewed by two others. Descriptive outcomes such as participant sample size, sex, age, experimental design, intervention and control task, and duration were extracted. Any data indicative of (neuro)physiologic assessment of both mental fatigue and control interventions, pre-, during, and post-intervention were also obtained and standardized mean difference (SMD); Hedges *g* and 95% confidence interval (95% CI) was calculated using the “esc” package in R studio (build 421, R Core Team). For studies incorporating a within-subject design, SMD was calculated depending on the data available, e.g., post-intervention data or final timepoint of intervention. Where a between-subjects intervention was used, in order to minimize bias that could arise due to differences in baseline outcomes, SMD was based on the mean gain score (e.g., pre-post intervention change) of the mental fatigue and control conditions, respective group n, correlation values between pre- and post-intervention data, and either the standard deviation (SD) of the gain or pre- and post-intervention SD. If data were unavailable to determine the correlation value, this was imputed from other studies with sufficient information. If data for between-subject studies could not adopt this method, SMD was estimated using the same approach for within-subject designs.

### Statistical analyses

Meta-analysis was performed (SPSS v29, IBM Corporation, USA) using a generic inverse variance, random- effects model with 95% CI. The restricted maximum likelihood estimator was used, and Knapp–Hartung standard error adjustments were applied where appropriate. Significance was investigated using *P* values, where alpha was set at ≤ 0.05. SMD was calculated and reported as Hedges (*g*) to reduce bias from small studies (Borenstein et al., 2021). The magnitude of the effect was determined using standardized conventions and represented as trivial (< 0.20), small (0.20–0.49), moderate (0.50–0.79), and large (≥ 0.80) (Borenstein et al., 2021). Between-studies heterogeneity was investigated using Cochrane’s *Q*, where alpha was set at *P* ≤ 0.10. The *I*^2^ statistic was used to determine the magnitude of heterogeneity and graded as low (0–40%), moderate (30–60%), substantial (50–90%), and considerable (≥ 75%) (Higgins et al., 2019). Subgroup analysis was conducted to explore the effects of duration (< 30 and ≥ 30 min), type of control or intervention task, or different methods of outcome occurrences (e.g., saliva vs. plasma). Additionally, subgroup analysis was conducted where multiple data points were discovered that were related. Specifically, analysis was conducted using representative effect sizes that were the most (closest to the null finding; CON) and least conservative (most pronounced effects; LC) of the available datasets. The latter was also extended where appropriate to include datasets that reflected the most negative (LC-) and positive (LC+) datasets. Meta-regression was conducted where appropriate to determine whether intervention task duration may be related to outcomes. Procedures of these analyses were consistent with published norms (Borenstein et al., 2021). Publication bias was examined statistically using eggers regression- based test (Borenstein et al., 2021). Where bias was detected Duval and Tweedie’s trim and fill correction was applied and the resultant effects on Hedges *g* and the 95% CI were considered. Where fewer than three studies were present, meta-analysis was not performed, and qualitative synthesis was considered.

## Results

### Included study characteristics and outcome measures of mental fatigue

Of the 72 studies included in this review, independent data sets existed in 14 studies (Behrens et al., 2018; Clark et al., 2019; Dallaway et al., 2022; Hess & Ennis, 2012; Klaassen et al., 2013; Kowalski et al., 2022; Le et al., 2021; Lopes et al., 2020; Martin et al., 2016; Mlynski et al., 2021; Van Cutsem et al., 2019; Wright et al., 2003, 2007, 2008), and non-independent subgroups occurred in seven citations (Filipas et al., 2018; Gantois et al., 2020; O'Keeffe et al., 2020; Rozand et al., 2014; Schücker & MacMahon, 2016; Smith et al., 2019; Widyanti et al., 2017). Supplementary Table 1 (https://osf.io/97xad/) outlines all citations and any subgroups. Across all datasets, (neuro)physiologic data were obtained from a total of 2,364 participants, of which 49.2% and 45.3% were male and female, respectively, while the remaining 5.5% were not reported. Most studies examined young to middle-aged adults (*n* = 66; range between 18.9 and 41.7 years), three studies (Filipas et al., 2018; Moreira et al., 2018; Penna et al., 2018) examined children and adolescents (range between 11.0 and 15.5 years), three studies (Behrens et al., 2018; Hess & Ennis, 2012; Klaassen et al., 2013) investigated middle-aged to older adults (range between ~ 50.0 and 72.0 years), and some did not report age (Dang et al., 2016; Head et al., 2017; Wright et al., 2003, 2007, 2008). A within-subjects design was used by most (*n* = 59) and the remaining 13 incorporated independent groups.

Supplementary Table 1 (https://osf.io/97xad/) summarizes intervention methods. The most popular approach was the Stroop task (*n* = 38). The color variant was incorporated on nine occasions, with most using 100% trial incongruency (Brown & Bray, 2019; Dallaway et al., 2022; de Lima‐Junior et al., 2023; Fairclough & Houston, 2004; Filipas et al., 2018), however, 75% (Salihu et al., 2023), 70% (Le et al., 2021), and 50% were also used (Dang et al., 2016; Goudini et al., 2024; Smith et al., 2019). The word version was also employed (Gantois et al., 2020), and some did not report intervention details (Hakim et al., 2022). A mixed Stroop task was used on the remaining occasions (*n* = 27), where the color variant was adopted, but one color was nominated to correspond to the word variant. Three studies (Habay et al., 2021a, 2021b; Proost et al., 2024; Van Cutsem et al., 2022) advanced this by also adding individualized time pressure on participants to respond (e.g., reduced presentation duration of stimuli). The AX-CPT was used on 11 occasions (Brownsberger et al., 2013; MacMahon et al., 2019; Marcora et al., 2009; Mlynski et al., 2021; O'Keeffe et al., 2020; Pageaux et al., 2013; Smith et al., 2015). The 2-back test was incorporated in six instances (Shigihara et al., 2012, 2013; Tanaka et al., 2012; Wylie et al., 2017), while Matuz et al. (2021) adapted this to include audio-visual stimuli (Gatekeeper task). Arithmetic tasks were adopted on seven occasions, and included serial subtraction (Gieseler et al., 2021; Hess & Ennis, 2012; Wright et al., 2003, 2007), addition (Galy & Mélan, 2015), and arithmetic and logic questions (Filipas et al., 2018; Park et al., 2021). The PVT was used on two occasions (Kowalski et al., 2022; Smith et al., 2019). While some (Holgado et al., 2023a, 2023b; O'Keeffe et al., 2020) administered the TloadDback task which introduced individualized time pressure. Four studies adopted Go/no-go like assessments, which included a conventional 10-min version of this task (Timme et al., 2022), or replacing words with numerical values (Head et al., 2016, 2017). Others (Behrens et al., 2018) modified the Go/no-go task to include a left and right handed stimulus that needed overriding when a tone occurred. Attentional and working memory tasks involving monitoring a single or multiple item counts (Jacquet et al., 2024; Widyanti et al., 2017), or noting letters next to a vowel (Wright et al., 2008) were also found. The rapid visual information processing task was used by Pires et al. (2018), while a switching (Budini et al., 2022), and transcription task (Englert et al., 2019) was also reported. Some studies (Clark et al., 2019; Klaassen et al., 2013; Otani et al., 2017) included several cognitive tasks as the intervention. One battery consisted of the color Stroop and N-back test (Clark et al., 2019). Another utilized a Stroop and N-back test, mental arithmetic, and puzzles (Klaassen et al., 2013). Finally, Otani et al. (2017) incorporated a Stroop, Sternberg memory test, and the rapid visual information processing task. Mental fatigue interventions ranged between 4 (Galy & Mélan, 2015) to 100 min (Budini et al., 2022).

The most common form of control intervention was media viewing (video control; *n* = 45). Less complex cognitive tasks were also used (*n* = 26). Alternative approaches included blank screen viewing (Martin et al., 2016), and painting (Filipas et al., 2018). Some allowed participants to view media or read magazines (Klaassen et al., 2013; Pires et al., 2018; Smith et al., 2016), while others (Batista et al., 2021; Moreira et al., 2018) asked participants to complete media viewing for 10 min, followed by 20 min of relaxation activity. One study (MacMahon et al., 2014) used video, but asked participants to complete the AX-CPT in the final 6 min of the control intervention. Control tasks ranged from 4 (Boat et al., 2021; Galy & Mélan, 2015) to 100 min (Budini et al., 2022).

### Risk of bias

A total of 234 risk-of-bias appraisals were completed. Summary figures can be found online (https://osf.io/97xad/) and have been separated by study design (independent and crossover) for each respective outcome measure we identified. Table 1 summarizes the overall risk-of-bias findings with respect to outcome.

**Table 1:** Discovered outcomes and summary of overall risk of bias

Outcome	Observations	Low bias	Some concerns (*n*)	High risk (*n*)
Heart rate	49	0	28 (569)	21 (341)
High-frequency heart rate variability	8	0	5 (96)	3 (47)
Low-frequency heart rate variability	12	0	5 (222)	7 (61)
Ratio of high- to low-frequency heart rate variability	7	0	4 (75)	3 (47)
RMSSD	15	0	7 (222)	8 (137)
SDNN	6	0	4 (91)	2 (40)
PNN50	6	0	2 (32)	4 (76)
R-R interval	2	0	1 (32)	1 (16)
Diastolic blood pressure	13	0	7 (250)	6 (140)
Systolic blood pressure	14	0	7 (250)	7 (319)
Mean arterial pressure	11	0	3 (96)	8 (148)
Cortisol	8	0	0	8 (123)
Glucose	13	0	4 (83)	9 (165)
Lactate	15	0	6 (92)	9 (102)
Galvanic skin conductance	4	0	0	4 (132)
Functional near-infrared spectroscopy	4	0	1 (22)	3 (37)
Electroencephalography	10	0	8 (118)	2 (26)
Functional magnetic resonance imaging	3*	0	0	3* (61)
Magnetoencephalography	3	0	0	3 (31)
Transcranial magnetic stimulation	6	0	6 (99)	0
Gaze fixations	2	0	0	2 (124)
Gaze fixation order	1	0	0	1 (12)
Pupil diameter	2	0	2 (83)	0
Eye blink rate	1	0	1 (84)	0
Capillary oxygen saturation	1	0	1 (29)	0
Pre-ejection period	3	1 (179)	2 (98)	0
Very low-frequency heart rate variability	3	0	3 (51)	0
Triangular index heart rate variability	1	0	1 (20)	0
Triangular interpolation heart rate variability	1	0	1 (20)	0
Poincaré cloud SD1 (heart rate variability)	1	0	1 (20)	0
Poincaré cloud SD2 (heart rate variability)	2	0	2 (41)	0
Poincaré cloud SD1/SD2 ratio	1	0	1 (20)	0
Breathing rate	1	0	1 (20)	0
Oxygen consumption	1	0	1 (18)	0
Salivary testosterone	1	0	0	1 (32)
Salivary alpha-amylase	1	0	0	1 (32)
Plasma sodium	2	0	0	2 (20)
Plasma potassium	2	0	0	2 (20)
Cutaneous vascular conductance	1	0	0	1 (8)
Totals	234	0	115	121

^*^ was provided for the functional magnetic resonance category to identify that the studies by Klaassen (13, 14, and 16) only account for one of these contributions. In column headings (*n*) represents the participant sample for the risk of bias assessment. *RMSSD* is the root mean square of successive differences, *SD* is standard deviation, *SDNN* is standard deviation of normal-to-normal intervals, *PNN50* is percentage of successive RR intervals that differ by more than 50 ms

## Quantitative synthesis

### Heart rate

**Fig. 2:**
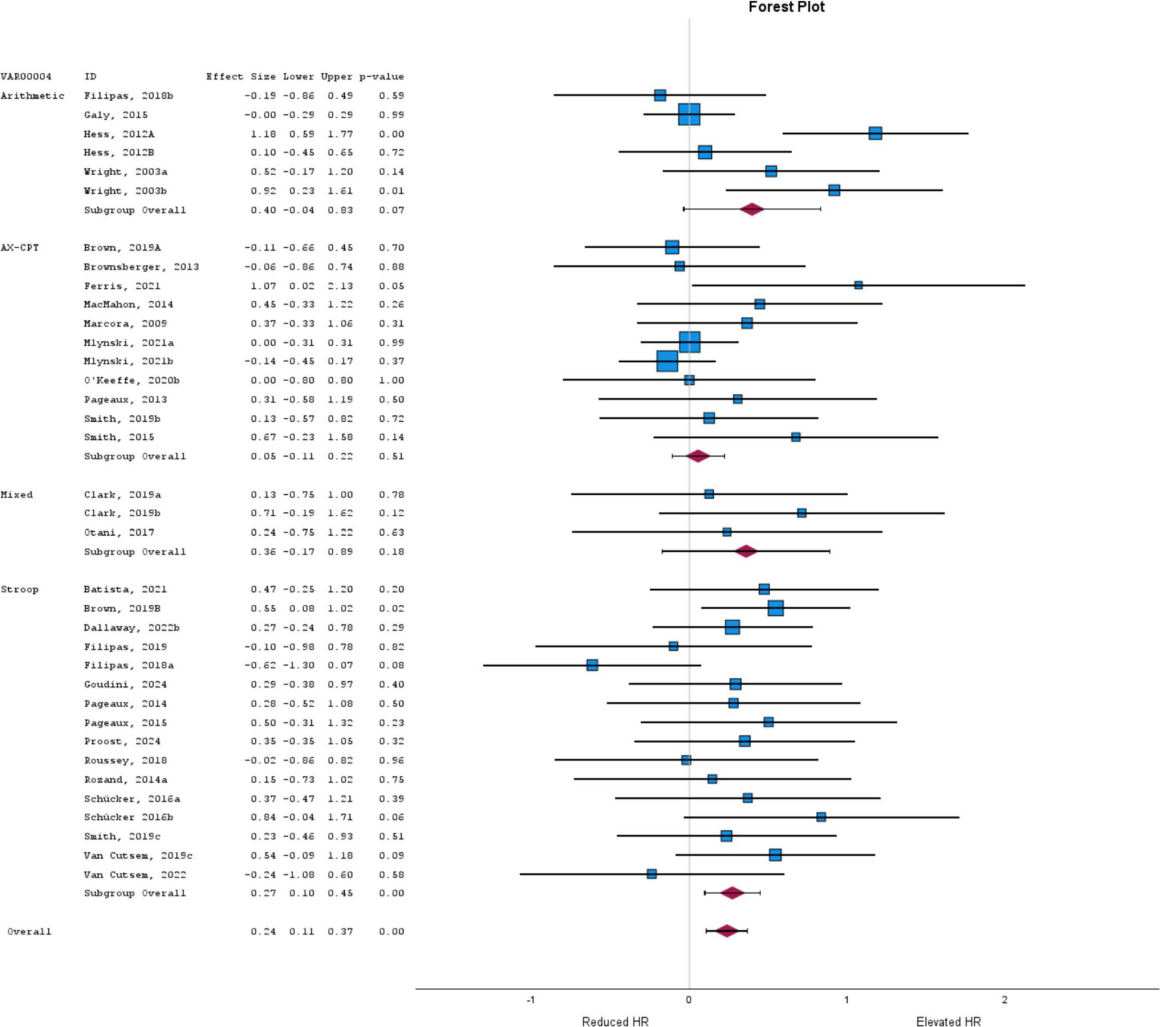
Subgroup analysis on heart rate (HR) based on the type of task used to induce mental fatigue. The ‘mixed’ subgroup consists of citations that applied multiple cognitive tasks for participants to complete in the allotted time. The size of the *blue squares* is proportional to the weight of the study. *Red diamonds* represent the subgroup effect. *Error bars* are 95% confidence intervals. Contributions from several studies (Budini et al., 2022; Dallaway et al., 2022; Head et al., 2016; O'Keeffe et al., 2020; Smith et al., 2019; Wright et al., 2003, 2008) were excluded as there was an insufficient number of studies to conduct quantitative synthesis on respective subgroups. Supplementary Table 1 (https://osf.io/97xad/) describes the interventions these excluded studies incorporated

Mental fatigue led to small elevations in HR (*g* = 0.23; 95% CI = 0.12 to 0.33; *P* < 0.01). This data was homogenous (*P* = 0.32). A similar finding arose when the least conservative dataset was incorporated (*g* = 0.26; 95% CI = 0.14 to 0.37; *P* < 0.01), but data displayed low heterogeneity (*Q*(39) = 53.41; *P* = 0.06; *I*^2^ = 29%). When sub-grouped by study design, both within- and between-subject experiments led to small elevations in HR (both *g* = 0.23; *P* ≤ 0.02). The former was homogenous (*P* = 0.87), however, between-study designs demonstrated moderate to substantial heterogeneity (*Q*(11) = 23.03; *P* = 0.02; *I*^2^ = 54%). When grouped by intervention task, the Stroop task elicited small HR elevations (*g* = 0.27; 95% CI = 0.10 to 0.45; *P* < 0.01; Fig. 2). This finding remained after replacing data with least conservative estimates (*g* = 0.31; 95% CI = 0.13 to 0.50; *P* < 0.01). Both were homogenous (both *P* ≥ 0.24). In the remaining tasks, mental fatigue and control were similar (*g* ranging from 0.05 to 0.36; all *P* ≥ 0.07). When separating data by control intervention, both video and cognitive tasks led to small elevations in HR (*g* = 0.29 and 0.27; both *P* ≤ 0.01). These findings were upheld when data were replaced with least conservative estimates (*g* = 0.31 and 0.27; both *P* ≤ 0.01). Although tasks incorporating a video control task displayed homogeneity (*P* = 0.74), cognitive task controls demonstrated moderate to substantial heterogeneity (*Q*(13) = 25.60; *P* = 0.02; *I*^2^ = 55%). When sub-grouped by intervention duration (e.g., < 30 and ≥ 30 min), small elevations in HR were observed irrespective of whether the most or least conservative data were included (*g* ranging from 0.21 to 0.38; all *P* < 0.01). Data were homogenous for the ≥ 30-min analyses (both *P* ≥ 0.83), but substantial heterogeneity existed for < 30 min (both *P* < 0.01; *I*2 between 60 and 68%). Meta-regression indicated there was no relationship between duration and HR (*t* = – 0.46; *P* = 0.65). Publication bias was not found for HR (*t* = – 0.85; all *P* = 0.40).

### Measures of heart rate variability

Meta-analyses were conducted on high and low frequency, the ratio between high and low frequency, SDNN, RMSSD, R-R interval width, and PNN50. Summary data is provided in Table 2. Irrespective of whether the most or least conservative data were incorporated, mental fatigue did not influence high-frequency power (both *P* ≥ 0.21), and there was no difference in subgroup analyses for this outcome (*g* between – 0.25 and 0.24; all *P* ≥ 0.21), except for the video control subgroup using the least conservative dataset (*g* = 0.40; 95% CI = 0.05 to 0.75; *P* = 0.02). A small increase in low-frequency power was apparent in mental fatigue when the most or least conservative data were adopted (*g* = 0.22 and 0.30; both *P* ≤ 0.04). While the conservative dataset was homogenous (*P* = 0.64) the least conservative estimates displayed moderate to substantial heterogeneity (*Q*(8) =  16.67; *P* = 0.03; *I*^2^ = 50%). The remaining subgroups indicated mental fatigue was similar to control for low frequency (g ranging from 0.12 to 0.33; all *P* ≥ 0.09; Table  2), except the video control, which indicated mental fatigue elicited a small increase (*g* = 0.28; 95% CI = 0.00 to 0.57; *P* = 0.05). For the ratio between high and low frequency, there was no difference between mental fatigue and control, irrespective of the data incorporated or subgroup examined (all *P* ≥ 0.54). Mental fatigue led to a small decline in SDNN irrespective of whether the most or least conservative data were considered (*g* = – 0.33 and – 0.35; both *P* ≤ 0.04). Both findings were homogenous (both *P* ≥ 0.19). This finding was identical in the video control and ≥ 30-min subgroup analyses, while the remaining subgroups indicated mental fatigue and control were similar (*g* ranging from – 0.26 to – 0.15; all *P* ≥ 0.09). A small increase in RMSSD was found when the most conservative estimates were incorporated (*g* = 0.25; 95% CI = 0.05 to 0.45; *P* = 0.01). Including the least conservative estimates resulted in a comparable RMSSD (*g* = 0.20; 95% CI = – 0.01 to 0.42; *P* = 0.07). Small to moderate increases in RMSSD were found in studies incorporating a between-subjects design, a Stroop task, and video control task interventions (*g* ranging from 0.25 to 0.41; all *P* ≤ 0.05). Studies adopting interventions longer than 30 min did not alter RMSSD (*g* = 0.14; 95% CI = – 0.07 to 0.36; *P* = 0.19). All analyses for RMSSD were homogenous (all *P* ≥ 0.15), except for the < 30-min duration subgroup, which showed moderate to substantial heterogeneity (Q(3) = 10.60; *P* = 0.01; *I*^2^ = 73%). R-R interval width and PNN50 were unaffected by mental fatigue irrespective of the data set used, or. subgroups examined (*g* ranging from 0.06 to 0.28; all *P* ≥ 0.12), and data were homogenous (all *P* ≥ 0.23). Across all heart rate variability outcomes, there was insufficient data to conduct quantitative synthesis in several subgroups (Table 2). None of the analyses demonstrated publication bias (all *P* ≥ 0.12).

**Table 2:** Effects for heart rate variability data

			Effect size			Heterogeneity test		
Outcome	Subgroup (sensitivity analysis)	*n*	*g*	95% CI	*P*	*Q*	*P*	*I*^2^ (%)
High frequency	Overall (CON)	6	0.02	– 0.33 to 0.37	0.91	8.41	0.14	42
	Overall (LC-)	6	– 0.07	– 0.48 to 0.34	0.74	**11.51**	**0.04**	**56**
	Overall (LC +)	6	0.24	– 0.13 to 0.61	0.21	**9.45**	**0.09**	**47**
	Between-subjects design	1						
	Within-subjects design	5	– 0.07	– 0.46 to 0.32	0.73	6.70	0.15	42
	2-back test	3	0.10	– 0.57 to 0.78	0.77	**7.22**	**0.03**	**74**
	AX-CPT	1						
	PVT	1						
	Stroop (CON)	3	– 0.25	– 0.67 to 0.17	0.24	1.02	0.60	0
	Stroop (LC)	3	0.01	– 0.51 to 0.52	0.98	2.97	0.23	34
	Cognitive task control	2						
	Video control (CON)	4	0.06	– 0.29 to 0.40	0.75	2.12	0.54	0
	Video control (LC-)	4	– 0.08	– 0.55 to 0.38	0.72	5.12	0.16	42
	Video control (LC +)	4	**0.40**	**0.05 to 0.75**	**0.02**	1.57	0.67	0
	< 30-min duration	0						
	≥ 30-min duration (CON)	6	0.02	– 0.33 to 0.37	0.91	8.41	0.14	42
	≥ 30-min duration (LC-)	6	– 0.07	– 0.48 to 0.34	0.74	**11.51**	**0.04**	**56**
	≥ 30-min duration (LC +)	6	0.24	– 0.13 to 0.61	0.21	**9.45**	**0.09**	**47**
Low frequency	Overall (CON)	9	**0.22**	**0.02 to 0.41**	**0.03**	6.04	0.64	0
	Overall (LC)	9	**0.30**	**0.01 to 0.59**	**0.04**	**16.67**	**0.03**	**50**
	Between-subjects design	1						
	Within-subjects design (CON)	8	0.21	– 0.03 to 0.39	0.09	4.92	0.67	0
	Within-subjects design (LC)	8	0.27	– 0.05 to 0.59	0.10	**16.34**	**0.02**	**54**
	2-back test	3	0.12	– 0.26 to 0.49	0.55	2.61	0.27	19
	AX-CPT	1						
	Go/no-go	2						
	PVT	1						
	Stroop (CON)	3	0.07	– 0.35 to 0.49	0.73	1.90	0.39	0
	Stroop (LC)	3	0.33	– 0.09 to 0.75	0.13	0.17	0.92	0
	Attentional task	1						
	Cognitive task control (CON)	3	0.15	– 0.21 to 0.51	0.42	2.96	0.23	36
	Cognitive task control (LC)	3	0.29	– 0.40 to 0.98	0.41	**12.16**	** < 0.01**	**82**
	Video control (CON)	6	0.24	– 0.04 to 0.53	0.09	2.99	0.70	0
	Video control (LC)	6	**0.28**	**0.00 to 0.57**	**0.05**	3.92	0.56	0
	< 30-min duration	0						
	≥ 30-min duration (CON)	8	0.14	– 0.09 to 0.37	0.23	4.63	0.71	0
	≥ 30-min duration (LC)	8	0.17	– 0.07 to 0.40	0.16	5.93	0.55	0
Ratio between high and low frequency	Overall (CON)	5	0.06	– 0.30 to 0.41	0.76	5.18	0.27	31
	Overall (LC-)	5	0.08	– 0.29 to 0.45	0.68	5.78	0.22	36
	Overall (LC +)	5	– 0.10	– 0.44 to 0.23	0.54	4.87	0.30	22
	Between-subjects design	0						
	Within-subjects design (CON)	5	0.06	– 0.30 to 0.41	0.76	5.18	0.27	31
	Within-subjects design (LC-)	5	0.08	– 0.29 to 0.45	0.68	5.78	0.22	36
	Within-subjects design (LC +)	5	– 0.10	– 0.44 to 0.23	0.54	4.87	0.30	22
	2-back	2						
	AX-CPT	1						
	PVT	1						
	Stroop	3	0.29	– 0.13 to 0.71	0.18	0.22	0.90	0
	Cognitive task control	2						
	Video control (CON)	3	0.29	– 0.13 to 0.71	0.18	0.22	0.90	0
	Video control (LC-)	3	– 0.01	– 0.47 to 0.45	0.97	2.32	0.30	15
	Video control (LC +)	3	0.32	– 0.10 to 0.74	0.14	0.45	0.80	0
	< 30-min duration	0						
	≥ 30-min duration (CON)	5	0.06	– 0.30 to 0.41	0.76	5.18	0.27	31
	≥ 30-min duration (LC-)	5	0.08	– 0.29 to 0.45	0.68	5.78	0.22	36
	≥ 30-min duration (LC +)	5	– 0.10	– 0.44 to 0.23	0.54	4.87	0.30	22
SDNN	Overall (CON)	5	**– 0.33**	**– 0.65 to – 0.02**	**0.04**	6.06	0.19	24
	Overall (LC)	5	**– 0.35**	**– 0.64 to – 0.07**	**0.01**	5.59	0.23	10
	Between-subjects design	2						
	Within-subjects design	3	– 0.26	– 0.92 to 0.40	0.44	**5.38**	**0.06**	**63**
	2-back	1						
	Go/no-go	1						
	Stroop	3	– 0.15	– 0.51 to 0.20	0.40	1.71	0.43	0
	Cognitive task control	0						
	Video control	5	**– 0.35**	**– 0.64 to – 0.07**	**0.01**	5.59	0.23	10
	< 30-min duration	1						
	≥ 30-min duration	5	**– 0.35**	**– 0.64 to – 0.07**	**0.01**	5.59	0.23	10
RMSSD	Overall (CON)	13	**0.25**	**0.05 to 0.45**	**0.01**	10.68	0.56	12
	Overall (LC)	13	0.20	– 0.01 to 0.42	0.07	14.08	0.30	24
	Between-subjects design	5	**0.41**	**0.04 to 0.78**	**0.03**	6.71	0.15	45
	Within-subjects design	8	0.10	– 0.16 to 0.36	0.43	1.37	0.99	0
	2-back	2						
	AX-CPT	1						
	Go/no-go	2						
	Stroop	8	**0.30**	**0.01 to 0.59**	**0.05**	7.88	0.34	26
	TloadDback	1						
	Cognitive task control	1						
	Video control	13	**0.25**	**0.05 to 0.45**	**0.01**	10.68	0.56	12
	< 30-min duration	4	0.23	– 0.35 to 0.81	0.44	**10.60**	**0.01**	**73**
	≥ 30-min duration	11	0.14	– 0.07 to 0.36	0.19	2.90	0.98	0
R-R interval	Overall	3	0.06	– 0.35 to 0.46	0.79	0.56	0.76	0
	Between-subjects design	1						
	Within-subjects design	2						
	2-back	1						
	Stroop	2						
	Cognitive task control	0						
	Video control	3	0.06	– 0.35 to 0.46	0.79	0.56	0.76	0
	< 30-min duration	0						
	≥ 30-min duration	3	0.06	– 0.35 to 0.46	0.79	0.56	0.76	0
PNN50	Overall (CON)	4	0.28	– 0.08 to 0.63	0.12	1.78	0.62	0
	Overall (LC)	4	0.16	– 0.27 to 0.58	0.47	4.33	0.23	30
	Between-subjects design	1						
	Within-subjects design	3	0.15	– 0.27 to 0.57	0.49	0.70	0.70	0
	2-back	1						
	AX-CPT	1						
	TloadDback	1						
	Stroop	2						
	Cognitive task control	1						
	Video control	4	0.28	– 0.08 to 0.63	0.12	1.78	0.62	0
	< 30-min duration	2						
	≥ 30-min duration	4	0.28	– 0.08 to 0.63	0.12	1.78	0.62	0

*AX-CPT* is AX-continuous performance task, *PNN50* is percentage of successive RR intervals that differ by more than 50 ms, *PVT* is psychomotor vigilance task, *RMSSD* is the root mean square of successive differences, and *SDNN* is standard deviation of normal-to-normal intervals. Where *CON* is stated, the data included in the analysis reflect those where *g* is closest to 0 (e.g., ‘most conservative’). Comparatively, *LC* is the ‘least conservative’ data set, and the positive and negative symbols indicate the dataset was least conservative in these respective directions. *Bolded* outcomes reflect significant findings. 95% confidence interval (CI) is the lower and upper bounds of the Hedges *g* estimate provided

### Measures of blood pressure

Quantitative synthesis was performed on diastolic and systolic blood pressure, and mean arterial pressure (Table 3). A small to moderate increase diastolic blood pressure occurred in mental fatigue (*g* = 0.47; 95% CI = 0.29 to 0.61; *P* < 0.01; Supplementary Fig. 2; https://osf.io/97xad/). All studies were between-subjects designs, so this analysis was identical to the overall effect. For arithmetic-based activities, mental fatigue resulted in a comparable increase in diastolic blood pressure (*g* = 0.43; 95% CI = 0.21 to 0.64; *P* < 0.01). Twelve studies used an intervention duration of < 30 min and a cognitive task control, these data also indicated a comparable elevation in diastolic blood pressure (*g* = 0.46; 95% CI = 0.29 to 0.63; *P* < 0.01). A small to moderate increase in systolic blood pressure resulted from mental fatigue (*g* = 0.40; 95% CI = 0.20 to 0.59; *P* < 0.01; Supplementary Fig. 3; https://osf.io/97xad/). All data were included exclusively from studies incorporating a between-subjects design.

Studies using arithmetic-based mental fatigue interventions produced small to moderate increases in systolic blood pressure (*g* = 0.43; 95% CI = 0.18 to 0.68; *P* < 0.01). Studies that incorporated interventions of < 30 min in duration also adopted a cognitive task as the control, resulting in small to moderate systolic blood pressure increases from mental fatigue (*g* = 0.41; 95% CI = 0.22 to 0.61; *P* < 0.01). Low to moderate heterogeneity was found for the overall effect, and the between-subjects and arithmetic subgroup analyses (all *P *≤ 0.07; *I*^2^ between 41 and 46). Compared to control, a small to moderate increase in mean arterial pressure in mental fatigue (*g* = 0.47; 95% CI = 0.28 to 0.65; *P* < 0.01; Supplementary Fig. 4; https://osf.io/97xad/).

An identical dataset was present for between-subjects designs, cognitive task control, and < 30-min intervention duration; indicating a comparable increase in mean arterial pressure (*g* = 0.48; 95% CI = 0.29 to 0.67; *P* < 0.01). For arithmetic-based mental fatigue interventions, mean arterial pressure also increased a small to moderate extent (*g* = 0.41; 95% CI = 0.16 to 0.67; *P* < 0.01). Across all pressure outcomes, there was insufficient data to conduct quantitative synthesis in several subgroups (Table 3). All analyses included homogenous data (all *P* ≥ 0.45). Publication bias was not found in any analysis (*t* ranging between – 0.49 and 1.36; all *P* ≥ 0.21).

**Table 3:** Effects for measures related to blood and arterial pressure

			Effect size			Heterogeneity test		
Outcome	Subgroup (sensitivity analysis)	*n*	*g*	95% CI	*P*	*Q*	*P*	*I*^2^ (%)
Diastolic blood pressure	Overall	13	**0.45**	**0.29 to 0.61**	** < 0.01**	4.92	0.96	0
	Between-subjects design	13	**0.45**	**0.29 to 0.61**	** < 0.01**	4.92	0.96	0
	Within-subjects design	0						
	Arithmetic	8	**0.43**	**0.21 to 0.64**	** < 0.01**	3.76	0.81	0
	Attentional task	2						
	AX-CPT	2						
	Switching attention	1						
	Cognitive task control	12	**0.46**	**0.29 to 0.63**	** < 0.01**	0.35	0.56	0
	Video	1						
	< 30-min duration	12	**0.46**	**0.29 to 0.63**	** < 0.01**	0.35	0.56	0
	≥ 30-min duration	1						
Systolic blood pressure	Overall	14	**0.40**	**0.20 to 0.59**	** < 0.01**	**21.64**	**0.06**	**41**
	Between-subjects design	14	**0.40**	**0.20 to 0.59**	** < 0.01**	**21.64**	**0.06**	**41**
	Within-subjects design	0						
	Arithmetic	9	**0.43**	**0.18 to 0.68**	** < 0.01**	**14.73**	**0.07**	**46**
	Attentional task	2						
	AX-CPT	2						
	Switching attention	1						
	Cognitive task control	13	**0.41**	**0.22 to 0.61**	** < 0.01**	20.67	0.37	42
	Video	1						
	< 30-min duration	13	**0.41**	**0.22 to 0.61**	** < 0.01**	20.67	0.37	42
	≥ 30-min duration	1						
Mean arterial pressure	Overall	11	**0.47**	**0.28 to 0.65**	** < 0.01**	4.19	0.94	0
	Between-subjects design	10	**0.48**	**0.29 to 0.67**	** < 0.01**	3.62	0.94	0
	Within-subjects design	1						
	Arithmetic	6	**0.41**	**0.16 to 0.67**	** < 0.01**	1.10	0.95	0
	Attentional task	2						
	AX-CPT	2						
	Mixed	1						
	Cognitive task control	10	**0.48**	**0.29 to 0.67**	** < 0.01**	3.62	0.94	0
	Video	1						
	< 30-min duration	10	**0.48**	**0.29 to 0.67**	** < 0.01**	3.62	0.94	0
	≥ 30-min duration	1						

*AX-CPT* is AX-continuous performance task. The ‘mixed’ subgroup consists of citations that applied multiple cognitive tasks for participants to complete in the allotted time. *Bolded* outcomes reflect a significant finding. 95% confidence interval (CI) is the lower and upper bounds of the Hedges *g* estimate provided

### Cortisol, glucose, and lactate

There were no significant changes in cortisol in any of the analyses conducted (*g* ranging from 0.04 to 0.28; all *P* ≥ 0.17). Similarly, mental fatigue did not influence glucose or lactate measures, irrespective of the analysis performed (*g* ranging from – 0.06 to 0.04; all *P* ≥ 0.68). Data in all analyses of cortisol, glucose, and lactate were homogenous (all *P* ≥ 0.20). There was insufficient data to conduct quantitative synthesis in several subgroups (Table 4). Publication bias was not detected in cortisol, glucose, and lactate analyses (*t* ranging between – 1.11 and 0.60; all *P* ≥ 0.31).

**Table 4:** Effects for plasma and salivary outcomes

			Effect size			Heterogeneity test		
Outcome	Subgroup (sensitivity analysis)	*n*	*g*	95% CI	*P*	*Q*	*P*	*I*^2^ (%)
Cortisol	Overall	7	0.09	– 0.21 to 0.39	0.56	6.75	0.34	17
	Plasma	2						
	Saliva	5	0.04	– 0.33 to 0.42	0.82	6.03	0.20	35
	Between-subjects design	0						
	Within-subjects design	7	0.09	– 0.21 to 0.39	0.56	6.75	0.34	17
	2-back	1						
	Mixed	4	0.28	– 0.12 to 0.69	0.17	1.43	0.70	0
	Stroop	2						
	Cognitive task control	1						
	Video	5	0.19	– 0.18 to 0.55	0.31	2.44	0.65	0
	< 30 duration	0						
	≥ 30-min duration	7	0.09	– 0.21 to 0.39	0.56	6.75	0.34	17
Glucose	Overall	5	0.04	– 0.33 to 0.40	0.85	0.27	0.99	0
	Between	0						
	Within	5	0.04	– 0.33 to 0.40	0.85	0.27	0.99	0
	AX-CPT	2						
	Stroop	3	0.01	– 0.46 to 0.47	0.98	0.13	0.94	0
	Cognitive task	0						
	Video	5	0.04	– 0.33 to 0.40	0.85	0.27	0.99	0
	< 30-min duration	0						
	≥ 30-min duration	5	0.04	– 0.33 to 0.40	0.85	0.27	0.99	0
Lactate	Overall	8	– 0.02	0.29 to 0.25	0.89	2.63	0.92	0
	Between	0						
	Within	8	– 0.02	0.29 to 0.25	0.89	2.63	0.92	0
	AX-CPT	2						
	Stroop	6	– 0.06	– 0.37 to 0.24	0.68	2.20	0.82	0
	Cognitive task	0						
	Video	8	– 0.02	0.29 to 0.25	0.89	2.63	0.92	0
	< 30-min duration	0						
	≥ 30-min duration	8	– 0.02	0.29 to 0.25	0.89	2.63	0.92	0

*AX-CPT* is AX-continuous performance task. The ‘mixed’ subgroup consists of citations that applied multiple cognitive tasks for participants to complete in the allotted time. 95% confidence interval (CI) is the lower and upper bounds of the Hedges *g* estimate provided

## Qualitative synthesis

### Blood and arterial pressure

Diastolic blood pressure was examined in seven studies with 13 independent subgroups valuated (Supplementary Table 1; https://osf.io/97xad/). All incorporated a between-subjects design, and mental fatigue interventions included arithmetic, attentional tests involving single- and dual-tasking, and the AX-CPT. All used a cognitive task control, except one who adopted video (Budini et al., 2022). Budini et al. (2022) found no difference between mental fatigue and control, whereas others (Mlynski et al., 2021; Wright et al., 2008) described condition effects where diastolic blood pressure was higher in mental fatigue. Due to the statistical approach taken by some (Hess & Ennis, 2012; Wright et al., 2003, 2007) it was difficult to ascertain whether condition effects may have existed. But based on data included on our analyses (Supplementary Fig. 2; https://osf.io/97xad/), one (of remaining eight) dataset suggested diastolic blood pressure was elevated by mental fatigue (Hess & Ennis, 2012). Collectively, mental fatigue increased diastolic blood pressure in three of the 13 subgroups.

Seven studies with 14 independent subgroups examined systolic blood pressure (Supplementary Table 1; https://osf.io/97xad/). First, Budini et al. (2022) reported no differences between groups. Mlynski et al. (2021) shared this outcome in one subgroup, but demonstrated significant systolic blood pressure elevations in the other. Both Gieseler et al. (2021) and Wright et al. (2008) reported elevation of systolic blood pressure due to mental fatigue. The approach used by some (Hess & Ennis, 2012; Wright et al., 2003, 2007) made it difficult to determine which subgroups demonstrated changes systolic blood pressure. But from the data incorporated into our analysis (Supplementary Fig. 3; https://osf.io/97xad/), two (of the remaining eight) subgroups suggested systolic blood pressure was elevated by mental fatigue (Hess & Ennis, 2012; Wright et al., 2007). Collectively, mental fatigue increased systolic blood pressure in five of the 14 subgroups examined.

Five studies examined mean arterial pressure (Mlynski et al., 2021; Otani et al., 2017; Wright et al., 2003, 2007, 2008). These studies are largely identical to those described above for blood pressure (replacing Budini et al. (2022) for Otani et al. (2017)). To extend this commentary, Otani et al. (2017) used a within-subjects design, with participants completing mixed battery of tasks, and a video control. These authors found mean arterial pressure was not effected by mental fatigue (Otani et al., 2017). Some reported increased mean arterial pressure (Mlynski et al., 2021; Wright et al., 2008). Examining our analysis (Supplementary Fig. 4; https://osf.io/97xad/) for subgroups from Wright et al. (2003) and Wright et al. (2007), only one demonstrated increased mean arterial pressure (Wright et al., 2003). Collectively, mental fatigue increased mean arterial pressure in three of the 11 subgroups examined. Lastly, half of the studies (4/8) examining blood and arterial pressure originated from the same research group; led by Wright (Mlynski et al., 2021; Wright et al., 2003, 2007, 2008).

### Salivary, plasma, and blood measures

Cortisol was examined on eight occasions across six studies (Supplementary Table 1; https://osf.io/97xad/). Salivary outcomes were reported five times (Campos et al., 2019; Klaassen et al., 2013; Moreira et al., 2018; Tanaka et al., 2012), while the remaining used plasma measures (Boat et al., 2021; Clark et al., 2019). All studies were within-participant designs, and mental fatigue interventions included the mixed Stroop task, 2-back test, and mixed cognitive tasks. Control interventions predominantly involved media viewing (video; 5/8 comparisons), screen viewing and relaxation (Moreira et al., 2018), 100% congruent Stroop (Boat et al., 2021), and 0-back task (Tanaka et al., 2012), All studies reported that cortisol was unaltered by mental fatigue, except for Klaassen et al. (2013), who highlight a significant increase in the retrospectively devised ‘fatigue responders’ group.

Glucose was measured in 12 studies (Supplementary Table 1; https://osf.io/97xad/). A within-subjects design was most commonly adopted (12/14), and two studies used independent groups (Fairclough & Houston, 2004; Rouse et al., 2013). Mental fatigue interventions consisted of variants of the Stroop task (10/14), the AX-CPT (2/12), and mixed tasks (Clark et al., 2019). Video controls were used on ten occasions, and cognitive tasks in the remaining. Across all contributions, mental fatigue failed to elicit significant glucose changes, except Fairclough and Houston (2004) who reported significant reductions in the change in blood glucose from baseline due to mental fatigue. Lactate was examined in 12 studies (Supplementary Table 1; https://osf.io/97xad/). A within-subjects design for each. Variants of the Stroop task were most common (10/12), but the AX-CPT (MacMahon et al., 2014; Smith et al., 2015), and a mixed battery (Clark et al., 2019) were also used. Video control was used in 11 instances, and a 100% congruent Stroop task (Pageaux et al., 2014), and screen starting (Martin et al., 2016) were also incorporated. Mental fatigue did not significantly alter lactate concentration on any occasion.

### Other physiologic measures

Several other physiologic and cardiovascular outcomes were identified. These included galvanic skin conductance (O'Keeffe et al., 2020; Widyanti et al., 2017), very low-frequency heart rate variability (Smith et al., 2019), Poincaré cloud SD2 (Matuz et al., 2021; Van Cutsem et al., 2022) and SD1, and the ratio of SD1/SD2, triangular index and triangular interpolation, and breathing rate (Van Cutsem et al., 2022), oxygen consumption (Head et al., 2016), salivary testosterone and alpha-amylase (Moreira et al., 2018), plasma sodium and potassium concentration (Clark et al., 2019), capillary oxygen saturation (Budini et al., 2022), pre-ejection rate (Gieseler et al., 2021; Mlynski et al., 2021), and cutaneous vascular conductance (Otani et al., 2017). Methodologies incorporated for these studies are reported in Supplementary Table 1 (https://osf.io/97xad/). Across each, mental fatigue did not differ from control, except galvanic skin conductance (O'Keeffe et al., 2020), capillary oxygen saturation (Budini et al., 2022), and pre-ejection period (Mlynski et al., 2021).

### Functional near-infrared spectroscopy

Three studies employed fNIRS to investigate hemodynamic responses to mental fatigue (Angius et al., 2022; Clark et al., 2019; Holgado et al., 2022). Among these, the left prefrontal cortex (probes placed at Fp1, or between Fp1 and F3) was the primary target of each investigation. Most showed no change in oxygenated, deoxygenated, and total hemoglobin concentration (Clark et al., 2019; Holgado et al., 2022), or the tissue oxygen index (Clark et al., 2019). However, Angius et al. (2022) found mental fatigue increased left PFC activity, as evidenced by elevated oxygenated and total, and reduced deoxygenated hemoglobin concentrations.

### Electroencephalography

All studies except Park et al. (2021) examined spectral band outcomes from EEG in response to interventions. Only one study (Jacquet et al., 2024) reported the gamma power spectrum (30–40 Hz), but these authors did not report data or analysis of this outcome. Ratios between spectral bands (theta/beta and theta/alpha ratios) were also examined by Tanaka et al. (2012). Although a condition effect was absent, these authors reported the ratio of theta/beta activity increased pre- to post-intervention in mental fatigue but not control, indicating a time-on- task effect (Tanaka et al., 2012). Some also investigated event-related potentials, summarized in Table 5.

**Table 5:** Summary of event-related potential findings

Citation	Event-related potential	Position (10–20 coordinates)	Amplitude	Latency
Habay et al., (2021a)	N1 (90–150 ms)	F3, Fz, F4	**↓**	↔
		FC1, FC2	**↓**	↔
		C3, Cz, C4	**↓**	↔
	P2 (80–260 ms)	F3, Fz, F4	↔	**↑**
		F7	↔	**↑**
	N2 (200–315 ms)	F3, Fz, F4	↔	↔
		F7	↔	↔
	P3b (280–450 ms)	P3, P4	↔	↔
		P7, P8, PO9, PO10	**↓**	↔
		Pz	↔	↔
Jacquet et al. (2024)	P1 (100–160 ms)	O1, O2	↔	Not reported
	N1 (160–220 ms)	P7, P8	**↓**	Not reported
Park et al. (2021)	P600 (530–750 ms)	F3, F4	↔	↔
		C3, C4	↔	↔
		P3, P4	↔	↔
		O1, O2	↔	↑ O1 only

Effects on amplitude and latency reflect how mental fatigue was influenced respectively to control

Delta activity (~ 1–4 Hz) was examined in three studies (Jacquet et al., 2024; Smith et al., 2019; Tanaka et al., 2012). Increases in this band at F3 and F4 were identified by Smith et al. (2019) in each of the experimental (PVT, AX-CPT, Stroop, and video control) conditions as time-on-task increased. However, delta power in these regions was elevated in the PVT and Stroop tasks compared to the control. In contrast to these findings, Tanaka et al. (2012) failed to identify any changes in delta activity (F3, Fz, F4, C3, Cz, C4, P3, Pz, P4, O1, and O2) between the 2-back and control 0-back task. Although highlighted by Jacquet et al. (2024), these authors did not report data or analysis of this spectral band.

Theta activity (~ 4–8 Hz) was monitored in six studies (Habay et al., 2021a, 2021b; Jacquet et al., 2024; Pires et al., 2018; Proost et al., 2024; Smith et al., 2019; Tanaka et al., 2012). Table 6 summarizes these outcomes.

**Table 6:** Changes in theta activity in the cited literature

Citation	Region	Position (10–20 coordinates)	Findings between interventions	Findings within intervention (time-on-task)
Habay et al., (2021a)	Dorsolateral prefrontal cortex	F3, Fz, F4	↔	↔
	Premotor cortex	FC1, FC2	↔	↔
	Primary motor cortex	C3, Cz, C4	↔	↔
	Inferior/ orbitofrontal cortex	F7	↔	↔
	Angular gyrus	P3, P4	↔	↔
	Fusiform gyrus	P7, P8, PO9, PO10	↔	↔
	Somatosensory/ association cortex	Pz	↔	↔
Pires et al. (2018)	Prefrontal cortex	Fp1	**↑**	↔
Smith et al. (2019)	Prefrontal cortex	F3, F4	↑ in PVT ↔ for Stroop and AX-CPT	↑ in PVT ↔ for Stroop and AX-CPT
Tanaka et al. (2012)	Dorsolateral prefrontal cortex*	F3, Fz, F4	↔	**↑**
	Primary motor cortex*	C3, Cz, C4	↔	↔
	Angular gyrus*	P3, P4	↔	↔
	Somatosensory/ association cortex*	Pz	↔	↔
	Not reported	O1, O2	↔	↔
Proost et al. (2024)	Inferior/ orbitofrontal cortex	F7	↔	↔
	Broca's area	FC6, F8	↔	↔
	Dorsolateral prefrontal cortex	F3, Fz, F4	↔	↔
	Anterior prefrontal cortex	FP1, FP2	↔	↔
	Premotor area	FC1, FC2	↔	↔
	Primary motor cortex	C3, Cz, C4	↔	↔
	Somatosensory association cortex	Pz	**↑**	↔
	Angular gyrus	P3, P4	**↑**	↔
	Fusiform gyrus	P7, P8, PO9, PO10	↔	↔
Jacquet et al. (2024)	Frontal region	AFz, AF3, AF4, Fz, F1, F2, F3, F4, F5, F6	↔	↔
	Central region	FCz, FC1, FC2, FC3, FC4, FC5, FC6, Cz, C1, C2, C3, C4, C5, C6, CPz, CP1, CP2, CP3, CP4, CP5, CP	↔	↔
	Parietal region	Pz, P1, P2, P3, P4, P5, P6, POz, PO3, PO4, Oz, O1, O2	↔	↔

Compared to the control, Pires et al. (2018) found that theta power was elevated at Fp1 in a rapid visual information processing task. Increased theta activity was also reported by Smith et al. (2019) at F3 and F4 in the PVT condition compared to the video control. These authors also report an theta elevation in these regions in the PVT as a product of time-on-task. Tanaka et al. (2012) highlighted a time-on-task effect at Fz in the 2-back test, but not the 0-back tasks. Proost et al. (2024) reported mental fatigue increased theta activity at the somatosensory association cortex (Pz), and angular gyrus (P3 and P4). These authors reported condition effects for the primary motor cortex (C3, Cz, and C4) and fusiform gyrus (P7, P8, PO9, PO10), but only for pre-intervention comparisons. Theta was unchanged in the remaining studies (Habay et al., 2021a, 2021b; Jacquet et al., 2024).

AX-CPT is the AX-continuous performance task, PVT is psychomotor vigilance test. * indicates alignment based on Habay et al., (2021a, 2021b). Tanaka et al. (2012) did not describe the corresponding region that coordinates align with Jacquet et al. (2024) grouped several coordinates together to form broader cortical representations.

Alpha activity (8–13 Hz) was monitored in most studies, and some subdivided into lower (8–10 Hz) and upper (10–13 Hz) ranges (Habay et al., 2021a, 2021b; Smith et al., 2019). Compared to control, most reported that mental fatigue did not alter alpha activity (Brownsberger et al., 2013; Habay et al., 2021a, 2021b; Proost et al., 2024; Smith et al., 2019; Tanaka et al., 2012), except two (Jacquet et al., 2024; Smith et al., 2019). Smith et al. (2019) found that mental fatigue increased lower and upper alpha power at F3 and F4 in the PVT and AX-CPT, while lower (but not upper) band activity also increased in the Stroop task intervention. Smith et al. (2019) also found that upper alpha band activity at F3 and F4 increased as a product of time-on-task in each of the cognitive tasks administered to induce mental fatigue (PVT, AX-CPT, and Stroop), but not in the control task. Jacquet et al. (2024) also reported elevations in the alpha spectrum about the broad frontal, central, and parietal regions they defined in contrast to control. Comparatively, Tanaka et al. (2012) identified a reduction in alpha band activity at O2 in the 2-back task.

Beta activity (13–30 Hz) was quantified in four studies (Brownsberger et al., 2013; Jacquet et al., 2024; Smith et al., 2019; Tanaka et al., 2012). Most did not observe a change in beta compared to the control (Smith et al., 2019; Tanaka et al., 2012), except Brownsberger et al. (2013) who identified increased power at F3 in mental fatigue. Beta activity was unaltered in most studies as a product of time-on-task (Brownsberger et al., 2013; Smith et al., 2019), but Tanaka et al. (2012) reported elevated beta activity at Pz as the 2-back test persisted.

Although highlighted by Jacquet et al. (2024), these authors did not report data or analysis of this spectral band.

## Magnetoencephalography, functional magnetic resonance imaging, transcranial magnetic stimulation, and visual responses

Extending the EEG findings, magnetoencephalography was also examined (Ishii et al., 2013a, 2013b; Shigihara et al., 2012, 2013). First, Ishii, Tanaka, Shigihara, et al. (2013b) report a 2-back task resulted in a reduction in visual cortex alpha activity. These authors did not identify a change in any of the other bandwidths (theta, beta, or gamma bands). Secondly, Shigihara et al. (2013) examined alpha and beta bands, and report that alpha power reduced at the middle and right superior frontal gyrus in 2-back task compared to the 0- back control. Changes in beta band activity were also lower in the left pre-central gyrus in the mental fatigue condition compared to control (Shigihara et al., 2013). Several time-on-task effects were also identified in mental fatigue, whereby alpha power decreased at the middle frontal and superior frontal gyrus, increased at the inferior parietal lobule, superior parietal lobule, parahippocampal gyrus, uncus, postcentral gyrus, middle frontal gyrus, and inferior frontal gyrus. In the control, declines in alpha band occurred at the angular gyrus, and increased at the middle temporal, superior temporal, postcentral, superior frontal, inferior frontal, and medial frontal gyrus were produced. For beta power, mental fatigue reduced activity at the superior temporal gyrus, while the middle temporal gyrus and inferior parietal lobule increased. In the control, an increase occurred at the middle temporal, superior frontal, cingulate, and precentral gyrus. Shigihara et al. (2013) report no change in delta and theta power. Shigihara et al. (2012) used magnetoencephalography to determine changes in the visual-evoked magnetic field, however, no differences were found between to 2-back and 0-back tasks.

Although five studies quantified neural activity using fMRI (Klaassen et al., 2013, 2014, 2016; Van Cutsem et al., 2022; Wylie et al., 2017), the studies by Klaassen incorporated a substantial portion of the same participants, and are summarized as one dataset. Here, participants were grouped as cortisol responders or non-responders, or young (25–35) and middle-aged (50–61) schoolteachers. In all instances, mental fatigue was elicited through a mixed battery of tasks, and a video or leisure control task was used. Following intervention, fMRI was applied during a verbal memory encoding and recognition task, or parametric Sternberg letter task. In cortisol non-responders, during encoding, there was greater activity in the right ventrolateral prefrontal cortex, temporo-parietal junction, bilateral superior parietal cortex, and the putamen in the mental fatigue condition (Klaassen et al., 2013). During recognition, cortisol non-responders elicited heightened activity in the right dorsolateral prefrontal cortex, bilateral ventrolateral prefrontal cortex, left posterior temporal cortex, right superior and bilateral inferior parietal cortex, left caudate, left putamen, and right thalamus due to mental fatigue (Klaassen et al., 2013). There were no changes in cortical function that arose in the control in either cortisol responder or non-responder groups (Klaassen et al., 2013). When the letter Sternberg task was employed, mental fatigue did not alter cortical function. However, there was an interaction in load, fatigue and age, where an increase in dorsomedial prefrontal cortex occurred during higher load situations in mental fatigue for younger participants, but declined in the middle-older group (Klaassen et al., 2014).

Additionally, the right anterior cingulate cortex declined in activity in mental fatigue irrespective of age group (Klaassen et al., 2016). In their study, Van Cutsem et al. (2022) applied fMRI during a flanker task that was administered prior and following the Stroop task and video interventions. These authors found reduced blood– oxygen-level-dependent signal at the supramarginal gyrus, somatosensory association cortex, anterior cingulate cortex, corpus collosum, lateral ventricle, and right cerebrum in mental fatigue. Wylie et al. (2017) applied fMRI during a 2-back fatiguing intervention and 0-back cognitive control task. These authors specifically examined the operation of the anterior cingulate cortex, finding it became more active during mental fatigue, and less active in the control intervention.

Three studies incorporated transcranial magnetic stimulation to investigate corticospinal and function and cortex activation (Holgado et al., 2022; Kowalski et al., 2022; Salihu et al., 2023). First, Holgado et al. (2022) examined motor evoked potentials pre- and post-intervention (TloadDback task and 0-back control). These authors found that mental fatigue did not alter corticospinal excitability of the first dorsal interosseous muscle. This pathway was also examined by Salihu et al. (2023) who investigated motor evoked potentials, intracortical facilitation, and short and long intracortical inhibition prior to and following an incongruent Stroop task and video control intervention. However, these outcomes were not influenced by mental fatigue. Kowalski et al. (2022) examined corticospinal excitability of the tibulus anterior via the motor evoked potential in both male and females following a 30-min PVT or video control task. Irrespective of sex, these authors report mental fatigue did not alter corticospinal excitability. These authors also investigated the cortical silent period, finding that this lengthened in each condition and group, and was unlikely attributed to mental fatigue (Kowalski et al., 2022).

Five studies investigated visual responses to mental fatigue (Dang et al., 2016; de Lima‐Junior et al., 2023; Englert et al., 2019; Smith et al., 2016; Timme et al., 2022). The proportion of gaze fixations was monitored by Englert et al. (2019) after two groups of participants completed a 6-min transcription task. These authors then asked participants to maintain gaze on a target, finding fewer fixations in mental fatigue. Visual search behavior was investigated by Smith et al. (2016), after participants completed a 30-min Stroop task or magazine reading. These authors found that mental fatigue did not alter fixation number per second or fixation duration, but did identify a possibly lower effect on fixation order in mental fatigue, while the overall fixation percentage also appeared to decline (Smith et al., 2016). In their study, Timme et al. (2022) monitored pupil diameter during a go/no-go and a go/go task, but no differences were observed. Pupil diameter was also examined by de Lima‐Junior et al. (2023) following an incongruent Stroop and video control task, and the authors found diameter reduced in mental fatigue. Eye blink rate was investigated by Dang et al. (2016) during a post-intervention anti-saccadic task. These authors did not comment on whether mental fatigue altered this outcome, but through imputation of the provided figures, it is unlikely (*g* = – 0.15; 95% CI = – 0.58 to 0.27).

## Discussion

This review sought to investigate the breadth of (neuro)physiologic outcomes that have been used to evaluate mental fatigue, and determine whether these may be of use to investigators when researching this phenomenon. The subsequent discussion is divided into commentary on (i) physiologic outcomes, (ii) measurements of neurophysiologic and visual function, and (iii) approaches to inducing mental fatigue. Finally, we provide some methodologic suggestions for future researchers to consider when designing mental fatigue intervention studies.

## Indices of mental fatigue – physiologic

We identified 30 unique physiologic outcomes that have been incorporated to quantify mental fatigue. Across those we reviewed, we found that mental fatigue increased HR, diastolic and systolic blood pressure, and mean arterial pressure, while some indices of heart rate variability (e.g., increased low frequency and RMSSD, and reduced SDNN) were also altered. The use of HR to quantify mental fatigue was shown to be the most popular choice across the measures we examined (44% of all studies). Regulation of HR is achieved through the autonomic nervous system, via sympathetic and parasympathetic divisions. Engaging in complex cognitive workload is thought to stimulate the sympathetic division and subsequently may account for the observed increases (Lean & Shan, 2012). While this agrees with our findings, elevations in HR were not universally observed. Indeed, others (Delliaux et al., 2019; Matuz et al., 2021) report reductions in HR from time-on-task, which may be attributed to parasympathetic mechanisms. Our subgroup analysis indicated that the Stroop task (but not other tasks) elevated HR. One possibility is that this subgroup had greater statistical power due to more included studies (*n* = 16). For arithmetic-based interventions, data were approaching significance (*P* = 0.07; Fig. 2), and additional literature may support this finding. However, the AX-CPT contained a comparable sample to the Stroop task (*n* = 11). Based on the description above, one might infer the Stroop task requires greater cognitive work. However, this is unlikely as self-reported levels of difficulty or demand for these tasks are often comparable. For instance, after a 90-min AX-CPT, participants reported mental demand of 74.76 ± 21.50 on the National Aeronautics and Space Administration Task Load Index (Le Mansec et al., 2018). A similar range (75.28 ± 22.92) was reported by Staiano et al. (2018) following a 90-min Stroop task. Additionally, one study we reviewed (Smith et al., 2019) contained both AX-CPT and Stroop mental fatigue conditions (Smith, 2019b and c, respectively). As evidenced in Fig. 2 both demonstrate a comparable non- significant change in HR from mental fatigue. Likely, our observations may be attributed to the methodologic diversity between studies. For instance, the measurement of HR arose from peripheral pulse units, chest monitors, and ECG. Moreover, our approach to determining the effect sizes used in the analysis (e.g., comparison of final epoch, post-intervention, or change from baseline as appropriate) is likely a less holistic representation of the data and may have also contributed to these findings. Future analyses are needed to confirm whether some subgroups may be sensitive to eliciting HR change. Such approaches should consider implementing multi-level meta-analysis, as this may better encapsulate data provided at various timepoints (Kadlec et al., 2023).

Our findings concerning blood and arterial pressure also provide some prospective useful outcomes for researchers when exploring mental fatigue. These may also be influenced by autonomic nervous system changes in a complex manner and interact with compensatory influences in cardiac output and peripheral vascular resistance (Richter et al., 2008; Wright et al., 2003). While our analysis indicates these as promising findings, these conclusions were largely the product a single research group (Wright et al.). Some studies outside this group (Budini et al., 2022; Otani et al., 2017) indicate mental fatigue did not have a significant effect on diastolic blood pressure or mean arterial pressure (Supplementary Fig. 2 and 4, respectively; https://osf.io/97xad/). While others have produced supporting evidence (Gieseler et al., 2021; Hess & Ennis, 2012). Further studies are needed utilizing a wider variety of mental fatigue intervention types and methodologic approaches to explore their usefulness as objective markers of this phenomenon.

Advancing the insights shared above, heart rate variability is a technique permitting greater nuance into the investigation of autonomic function by examining beat-to-beat variability (Qin et al., 2021). Our analysis revealed that mental fatigue may influence several heart rate variability outcomes; e.g., reduced SDNN, and increased RMSSD and low-frequency power. Regarded as an overall representation of heart rate variability, our finding for SDNN may suggest that mental fatigue reduced overall variability (Kim et al., 2018; Tran et al., 2009). An elevation in low frequency may reflect an increase in sympathetic activity (Kim et al., 2018; Lean & Shan, 2012). Given mental workload may stimulate sympathetic nervous system activation to elevate HR, an increase in low frequency may be expected. However, a somewhat contradictory finding is our observation that RMSSD increased. This outcome is considered a marker of parasympathetic cardiac regulation or vagal tone, and its elevation suggests an increase in parasympathetic drive (Cao et al., 2019; Laborde et al., 2017). Recently, Matuz et al. (2021) monitored several heart rate variability outcomes related to vagal tone (e.g., RMSSD, pNN50, and high frequency) and found parasympathetic nervous system contributions to be a more dominant explanation of mental fatigue following a 90-min modified 2-back test. Evidently, this emphasizes that interventions may uniquely evoke changes in heart rate variability characteristics. In our analysis, we found heightened low- and high-frequency power, and reduced SDNN when control tasks incorporated video, but not a similar cognitive task. One explanation for this may be that cognitive control tasks evoke sympathetic nervous system changes that track closely to the complex (mentally fatiguing) task being undertaken, than a video control might. Hence, the nature of the control task may be a significant factor in producing heart rate variability changes. Due to insufficient data across several heart rate variability subgroup analyses, it is difficult to affirm what these methodologic characteristics may be; warranting further exploration. An alternative perspective from a recent systematic review (Csathó et al., 2023) is that increased parasympathetic dominance may be unrelated to the increased activity of stress systems, but rather, indicate disengagement from a task. Overlaying these findings with behavioral data such as task response time, accuracy, or lapses in attention may help to further distinguish this. As a technology, heart rate variability has been suggested to not be suitable for the detection of mental fatigue (Kunasegaran et al., 2023). Although we agree confounding influences could moderate its application (see Laborde et al. (2017) for a comprehensive review). From our analysis, it remains too early to exclude this technology and researchers should consider its application to investigate nuanced aspects of autonomic function that might moderate mental fatigue.

In addition to engaging sympathetic nervous system activity, cognitively demanding tasks have also been thought to elicit cortisol stress responses, which can be examined through blood or saliva (Bohnen et al., 1990; Kirschbaum & Hellhammer, 1994). Moreira et al. (2018) describe that mental fatigue may evoke the hypothalamic–pituitary–adrenal axis, which in turn stimulates the secretion of cortisol and other hormones such as alpha-amylase. The recent narrative review by Kunasegaran et al. (2023) describes salivary cortisol levels as a reliable indicator of mental fatigue and may provide useful supplemental data alongside other monitoring technologies (e.g., smart phone use in driver fatigue). Our quantitative and qualitative analysis suggests it is premature to make such affirmations; finding no effects in favor of this consensus, and reporting only one study (Klaassen et al., 2013) that reported increased salivary cortisol in a post hoc devised ‘responders’ group. Other data of hormonal function (testosterone and alpha-amylase) was not found to be influenced by mental fatigue, but this was based on only a single study (Moreira et al., 2018). Additionally, glucose has previously been posited as a limited resource in self-control tasks (Gailliot et al., 2007), becoming depleted as task engagement occurs. Similar to others (Vadillo et al., 2016), we also failed to observe any quantitative or qualitative evidence supporting this, except for one study (Fairclough & Houston, 2004), which reported a greater decline from baseline in mental fatigue than control. Mental fatigue also did not alter lactate kinetics, but we suspect the frequency of reporting for this outcome may have been attributed to its prominence in sequential tasks involving physical performance. Collectively, these measures are simple to collect and analyze. However, based on the quantitative and qualitative evidence provided here, they are unlikely to be informative markers of mental fatigue.

## Indices of mental fatigue – neural

Neuronal function assessed by EEG was a popular choice among the studies reviewed. Previously, Tran et al. (2020) have suggested that mental fatigue may alter frontal, central, and posterior theta activity, and central and posterior alpha activity. The findings from our review partially agree. We found that some (Pires et al., 2018; Smith et al., 2019; Tanaka et al., 2012) reported altered theta bandwidth in frontal regions. However, this was not universal (e.g., Habay et al., (2021a, 2021b), and the Stroop and AX-CPT conditions in Smith et al. (2019)). Mental fatigue also did not alter alpha band activity within the parietal and occipital regions (Brownsberger et al., 2013; Habay et al., 2021a, 2021b; Smith et al., 2019), except Jacquet et al. (2024) who found increased alpha activation in these and the frontal region. Adding further complexity, one study (Tanaka et al., 2012) reported reduced alpha activation in mental fatigue in the occipital region. There may be several reasons explaining the divergent findings. First, 16 of the 21 studies synthesized by Tran et al. (2020) included simulated or real-time driving or flying. Our review excluded simulation-based activities in favor of acute cognitive tasks, as this review was geared towards researchers investigating mental fatigue in laboratory settings, rather than ecological application; though interestingly only one study (Tanaka et al., 2012) was common between these reviews.

Moreover, these settings may create divergent fatigue states (see discussion concerning active and passive fatigue in the “Approaches to inducing mental fatigue” subsection below). Secondly, the conclusions from Tran et al. (2020) were based predominantly on studies utilizing a cohort design. Alternatively, we included literature that employed two trial arms; an intervention and control employing a cognitively stimulating intervention.

Circadian and homeostatic processes are known to cause EEG fluctuations (Ishii et al., 2014; Klimesch, 1996), hence, we wished to reduce the effects such confounding may have. We also excluded studies that incorporated sleep deprivation, of which two (Caldwell et al., 2002; Perrier et al., 2016) were synthesized by Tran et al. (2020). Despite these considerations, we believe there is merit to incorporating EEG when evaluating mental fatigue, and researchers should consider its application. Further data is needed among studies incorporating the context framed by this review as well as further scrutiny of event-related potentials, which were only explored in three studies (Habay et al., 2021a, 2021b; Jacquet et al., 2024; Park et al., 2021), but there may be some promise in exploring the N1 outcome. Such insights may reveal important characteristics of EEG for researchers to investigate, and enhance understanding of associated neural underpinnings. It would be valuable for data from future studies to be quantitatively synthesized to elucidate this. Such an approach should employ the practices used by Tran et al. (2020) or consider multi-level meta-analysis (Kadlec et al., 2023). These approaches will better consider the evaluation of multiple comparisons (such as epochs within a mental fatigue or control interventions) compared to traditional approaches.

Cerebral hemodynamic function was also investigated through application of fMRI and fNIRS. Previously, Salihu et al. (2022) have evaluated the neural mechanisms of mental fatigue through activation likelihood estimation meta-analysis. Among their findings, these authors suggest mental fatigue may create an interplay between structures associated with the cognitive control network and default mode network of the brain (Broyd et al., 2009; Cole & Schneider, 2007). Interestingly, reduced activation of several right-lateralized cortical and sub-cortical structures that comprise the cognitive control network arose, e.g., dorsolateral prefrontal cortex, anterior cingulate cortex, dorsal premotor area, and pre-supplementary motor area. While structures in the default mode network such as the posterior cingulate cortex, precuneus, and inferior parietal lobe became more active (Salihu et al., 2022). As described above, the review context can be critical. Hence, extrapolating these findings to the present synthesis is challenging. Comparing the inclusion practices between this and the review by Salihu et al. (2022), both considered mental fatigue emerging from cognitive tasks, but this was the extent of overlap. The only other requirements for inclusion in the review by Salihu et al. (2022) was that (i) brain activity and/or function was examined using fMRI, positron emission tomography, fNIRS, or transcranial magnetic stimulation, and ii) that relationships with self-report mental fatigue were drawn. Consequently, a broad basis of literature including healthy populations, those with motor impairment (e.g., multiple sclerosis etc.), and those with addictive behaviors (e.g., cocaine addiction) were integrated. Moreover, most of the studies we identified applied fMRI pre- and/or post-intervention cognitive assessment. While, one (Wylie et al., 2017) examined anterior cingulate cortex activity as a function of time-on-task to the mental fatigue or control interventions, reporting an increase in activity due to mental fatigue. Despite these limitations, we have identified similar changes in some neural areas highlighted by Salihu et al. (2022), and suggest a potential residual effect of mental fatigue may exist in the brain. For instance, Klaassen et al. (2016) report a decline in activity within the right anterior cingulate cortex in both younger and middle-older aged teachers during memory encoding. These same participants also showed changes in activity in the right superior and bilateral inferior parietal cortex during the recognition phase of the Sternberg letter task (Klaassen et al., 2013). Similarly, others demonstrated reduced activity in the anterior cingulate cortex and right cerebrum during a post-intervention flanker task (Van Cutsem et al., 2022). Previously, the anterior cingulate cortex has been acknowledged as an important region associated with mental fatigue (Lorist et al., 2005; Smith et al., 2018), given its role in effortful mental processing and control, performance monitoring, and task perseverance (Carter et al., 1998; Croxson et al., 2009; Etkin et al., 2011). This site has also been hypothesized to become inhibitory during prolonged mental workload due to the accumulation of adenosine, and hinder upstream information processing (Martin et al., 2018). Within the neurocognitive framework of fatigue proposed by Muller and Apps (2019), fatigue is thought to arise in two-distinct ways. First, activity begins to decline in site-specific structures responsible for task performance. Second, the anterior cingulate, dorsolateral prefrontal cortex and anterior insula form a non-task-specific domain that appraise task-related benefits and internal state and moderate motivated behavior through the application of effort; each declining in activity as a product of sustained operation. Recently, Darnai et al. (2023) have provided supporting evidence for this model, reporting that mental fatigue led to a reduction in activity in task-specific neuroanatomy, and areas responsible for motivational and evaluation processes (e.g., insula, anterior cingulate cortex and dorsolateral prefrontal cortex). Additionally, these influences were reversed through monetary reward (i.e., enhancing behavioral-motivation by rebalancing task-related benefits and costs). The neurocircuitry involved with mental fatigue is still emerging, however, researchers seeking to explore these should consider focusing their attention towards structures such as those described above as these seem to be the most probable based on the citations presented here and growing literature consensus.

Although fMRI can provide excellent spatial insights into neuroanatomical function, it is expensive and less accessible to most. Moreover, the prolonged task durations associated with the context of mental fatigue may also present an ethical conundrum due to radiation dose. A prospective alternative might include the application of fNIRS. Despite its spatial resolution limitations, this technology may offer a useful means of exploring cortical activity related to mental fatigue. Among studies we reviewed, the left prefrontal cortex was the only site examined, and only one study (Angius et al., 2022) reported altered activity about this site. From this and the discussion above, we suggest researchers consider examining bilateral function should channel availability permit, as this will provide the necessary data for later quantitative synthesis. If pressed, researchers might consider examining right frontoparietal areas such as the dorsolateral prefrontal cortex, the superior and inferior parietal lobules, middle frontal, and temporal gyrus, as these appear more attuned to mental fatigue. We also identified literature employing transcranial magnetic stimulation. Some examined corticospinal excitability to the first dorsal interosseous muscle (Holgado et al., 2022; Salihu et al., 2023) and tibulus anterior (Kowalski et al., 2022), but demonstrated these pathways may be unaffected mental fatigue. Salihu et al. (2023) also explored intracortical facilitation, and short, and long intracortical inhibition, and Kowalski et al. (2022) examined the cortical silent period, but mental fatigue did not perturb these outcomes. Holgado et al. (2022) have recently synthesized literature evaluating corticospinal excitability and mental fatigue and suggest that its current application has not positioned it to be an effective marker and greater methodological consistency is needed.

Based on our analysis, we would agree transcranial magnetic stimulation may not be a promising channel.

Of the visual function responses identified (gaze fixation and fixation order, pupil diameter, and blink rate), only one study (de Lima‐Junior et al., 2023) reported pupil diameter being reduced by mental fatigue. Eye-tracking and electro-oculography have been theorized to reflect this phenomenon due to outcomes demonstrating relationships to neural function and neurotransmitter operation (Eckstein et al., 2017; Jongkees & Colzato, 2016). For example, pupil dilation correlates with areas within the default mode network (Yellin et al., 2015), while eye-blink rate may reflect dopamine activity (Jongkees & Colzato, 2016). A recent systematic review (Bafna & Hansen, 2021) examined eye metrics and mental fatigue, and highlight mean and/or peak velocity saccade movements to be a useful monitoring tool. These authors provide a synthesis of the associated neural underpinnings of saccadic operation (e.g., governance by the superior colliculus and the role of omnipause neurons). Further research is needed to better understand upstream moderating mechanisms. Saccadic eye movements were also recently suggested as a potential marker for mental fatigue by Kunasegaran et al. (2023), but these authors explain outcomes may be obscured through frontal lobe processing. Incorporating measures of blink functionality should be approached with caution (Bafna & Hansen, 2021), as compensatory changes in blink suppression and reduced eye moisture content may conflate changes related to altered dopamine abundance (Bafna & Hansen, 2021; Eckstein et al., 2017). Holistically, eye metrics provide insight into the regulation of visual gaze and cue identification. Slowed visual regard is likely to play a prominent role in the features of behavioral outcomes, e.g., slowed response time etc. Thus, we agree with the proposition of others (Bafna & Hansen, 2021; Kunasegaran et al., 2023) that eye metrics may yet prove to be important objective measure of mental fatigue and researchers should consider their inclusion.

## Approaches to inducing mental fatigue

The second aim of this review was to evaluate the methodological characteristics of mental fatigue interventions examining (neuro)physiologic outcomes. Several cognitive tasks have been used to induce mental fatigue, including variations of the Stroop task, AX-CPT, N-back tests, and arithmetic. Largely, the cognitive task incorporated did not influence the interpretation of outcomes, except for the Stroop task and HR, and arithmetic in systolic, diastolic blood pressure, and mean arterial pressure; which all demonstrated subgroup effects.

However, in these instances, a substantiative portion of the data for these came from interventions employing such tasks. Further studies are required in order to gain a deeper appreciation of whether task specific differences manifest in (neuro)physiologic outcomes. Extending the discussion concerning intervention task type, some have sought to progress conventional approaches and individual parameters within the intervention (Habay et al., 2021a, 2021b; Holgado et al., 2023a, 2023b; O'Keeffe et al., 2020; Proost et al., 2024; Van Cutsem et al., 2022). Primarily, this involved integrating time pressures to increase information processing demands, which may be pivotal to inducing mental fatigue (Borragan et al., 2017). Foremost, those incorporating the TloadDback task (Holgado et al., 2023a, 2023b; O'Keeffe et al., 2020) and individualizing processing duration available for participants by manipulating the stimulus presentation period. During familiarization, the minimum duration needed for participants to maintain an accuracy in the task of 85% was determined and then incorporated into the mental fatigue intervention task. Others (Habay et al., 2021a, 2021b; Proost et al., 2024; Van Cutsem et al., 2022) have also applied this to the mixed Stroop task. Such approaches may prove to be a useful means for researchers to better manage individual variability that may exist between participants (Habay et al., 2023; MacMahon et al., 2023; Martin et al., 2016), or to leverage this to create more ecologically representative tasks. Of the control tasks used, videos commonly showing documentaries were the most popular selection. Second, were easier variants of the mental fatigue intervention task. As discussed above, the nature of the control task may be an important feature that moderates (neuro)physiologic outcomes. However, more data is needed across a range of control types to extend this understanding.

Regarding intervention duration, the most popular selection was 30 min, likely as a product of the proposed threshold of this duration suggested by Van Cutsem et al. (2017). Interestingly, this was closely followed by tasks lasting fewer than 11 min. Studies encompassing this latter duration were largely aligned to those within the ‘depletion literature’, where shorter duration tasks are more common (Brown et al., 2019). Also prevalent was the 90-min selection, where many (Brownsberger et al., 2013; O'Keeffe et al., 2020; Smith et al., 2015) appear to have cited the successes of early work on the phenomenon, e.g., Marcora et al. (2009), as a justification for nominating this duration. From our analyses concerning intervention duration (e.g., < 30- and ≥ 30-min subgroups and duration meta-regression), we agree with the position of Brown et al. (2019), who describe the ‘non-existence’ of a 30-min threshold. Also concerning duration, previous discussion has proposed two divergent mental fatigue states may exist (active and passive), each with nuanced underlying mechanisms (Hancock & Desmond, 2001; MacMahon et al., 2023; Pattyn et al., 2018). The former is thought to be the product of effortful cognitive engagement, whereas passive fatigue may arise when tasks are monotonous or boring (MacMahon et al., 2023; Pattyn et al., 2018). Though often attributed to workload (e.g., higher and reduced workload, respectively), task duration and task selection are likely important considerations. For instance, selecting longer duration tasks may evoke passive fatigue, the mechanisms for which may diverge from other approaches such as the individualized approaches described above; e.g., constraining information-processing duration may be governed differently to settings involving underload. Moreover, some designs may incorporate shorter control interventions (Martin et al., 2016), and may create instances where divergent fatigue states are triggered (passive vs. active fatigue). To reiterate the description of MacMahon et al. (2023), our understanding of these states is muddy and further research is indeed needed to better understand the active and passive state. Something that begins with researchers critically appraising the range of methodologic factors including the types of intervention and control tasks used, and important characteristics concerning these.

## Risk of bias and its future minimization

Of the outcomes evaluated, one study demonstrated low risk of bias (Gieseler et al., 2021) and high risk of bias was found in approximately half of the outcomes appraised. In order to minimize risk of bias and improve research quality the following should be considered. For Domain 1, researchers need to elaborate on the randomization process. Simply stating that a study was randomized is insufficient to conclude low risk of bias (Sterne et al., 2019). Indeed, the method and whether the allocation of the sequence was concealed until participants were assigned to their group or condition need to be described. For the latter, the assigner should remain unaware of the sequence. Importantly, in studies using a crossover design, investigators should report data from the first iteration of the study to determine the successfulness of randomization. For example, in a balanced crossover design, ten participants may be randomized to first complete the mental fatigue intervention, while the remaining ten complete the control. Researchers should describe relevant demographic and baseline characteristics of each ten participants and the condition they were allocated. Missing outcome data (Domain 3) was the most common source of overall high risk of bias (114 occasions; 96%); primarily due to insufficient detail reported to determine whether all data were included in respective analyses. Researchers should indicate the number of participants used either by directly reporting this in relevant tables/figures, or by correctly reporting statistical degrees of freedom. Alternatively, investigators should consider open-science practices and where possible, provide data in publicly available repositories. This approach has the added benefit for those conducting meta-analysis, as it reduces instances of missing data. Selection of the reported result (Domain 5) was often evaluated as ‘some concerns’, primarily due to our inability to discern whether results were analyzed in accordance with an a priori statistical analysis plan. Echoing the sentiments of Holgado et al., (2023a, 2023b), we would encourage investigators to provide sufficient methodological detail about experiments and pre-register this information in open access platforms such as Open Science Foundation (https://osf.io/) or clinical trial data bases if appropriate. Of the literature we evaluated, some (Gieseler et al., 2021; Holgado et al., 2023a, 2023b; Van Cutsem et al., 2022) employed this practice well and should be considered by others seeking to enact this recommendation.

## Conclusion

We sought to evaluate the range of (neuro)physiological outcomes used to examine mental fatigue in order to enhance monitoring practices of this phenomenon. Across those we identified, HR, some measures of heart rate variability (e.g., low frequency, SDNN, and RMSSD), systolic and diastolic blood, and mean arterial pressure, show promise and are worth further investigation. Moreover, neuroimaging techniques such as EEG and fNIRS may be useful tools to investigate cortical responses to mental fatigue. We found some evidence that mental fatigue may alter a range of bandwidths (e.g., increased delta, theta, alpha power) in frontal regions. Further application of fNIRS is required and right-lateralized frontoparietal structures should be considered by researchers. Despite the inclusion of 72 studies, further investigations are required that incorporate outcomes, across a range of methodologic factors. This review assists researchers investigating mental fatigue using (neuro)physiological outcomes, and provides a starting point to sift through the sleuth of factors available when designing interventions.

## Supplementary Information

Below is the link to the electronic supplementary material.

Supplementary file1 (DOCX 502 KB)

## Data Availability

The datasets generated during and/or analyzed during the current study are available via Open Science Framework, https://osf.io/97xad/.

## Code availability

There is no specific code related to this manuscript. Effect-size calculations were made using generic code using the “esc” package in R Studio.
