# Supplementary material for: Approaches to inducing mental fatigue: A systematic review and meta-analysis of (neuro)physiologic indices
Source: Behav Res Methods. 2025 Feb 26;57(4):102. doi: 10.3758/s13428-025-02620-7 (PMC11865143; doi:10.3758/s13428-025-02620-7)
Supplement: Supplementary file 1 — Supplementary file1 (DOCX 502 KB) [file 13428_2025_2620_MOESM1_ESM.docx]

Approaches to inducing mental fatigue: A systematic review and meta-analysis of (neuro)physiologic indices

Authors:

Goodman, S.P.J.a, Collins, B.b, Shorter, K.a,c, Moreland, A.T.d, Papic, C.e,a, Hamlin, A.S.a, Kassman, B.^a^,

Marino, F.E.f

a School of Science and Technology, University of New England, Armidale, NSW, Australia

b Holsworth Research Initiative, La Trobe Rural Health School, La Trobe University, Victoria, Australia

c Allied Health and Human Performance, University of South Australia, South Australia, Australia

d Re-MIND Institute, Sunbury, Victoria, Australia

^e^ RECOVER Injury Research Centre, Faculty of Health and Behavioural Sciences, The University of Queensland, Brisbane, QLD, Australia

f School of Rural Medicine, Charles Sturt University, Orange, NSW, Australia

Corresponding author:

Dr Stephen Goodman, [stephen.goodman@une.edu.au](mailto:stephen.goodman@une.edu.au)

Phone: 0267733345

Address: Room 251, Building S02, University of New England, Armidale, NSW, 2351, Australia

**
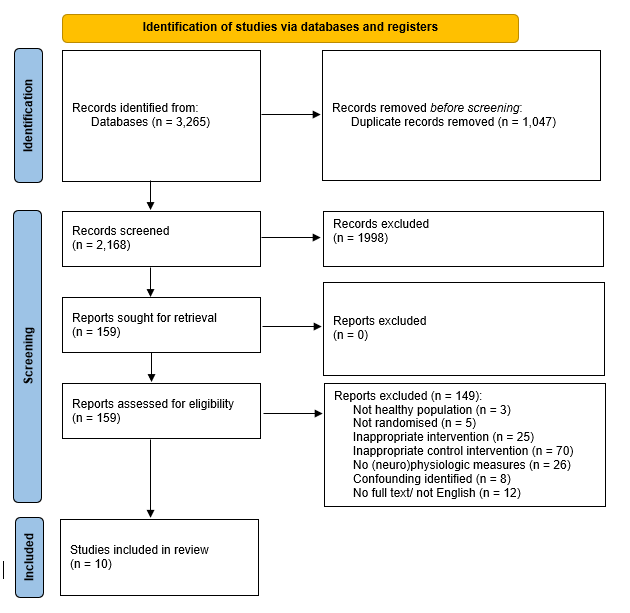
**

**Supplementary Figure 1.** Follow up literature search. Note the change to inclusion criteria to reflect the identification of literature examining (neuro)physiologic indices.


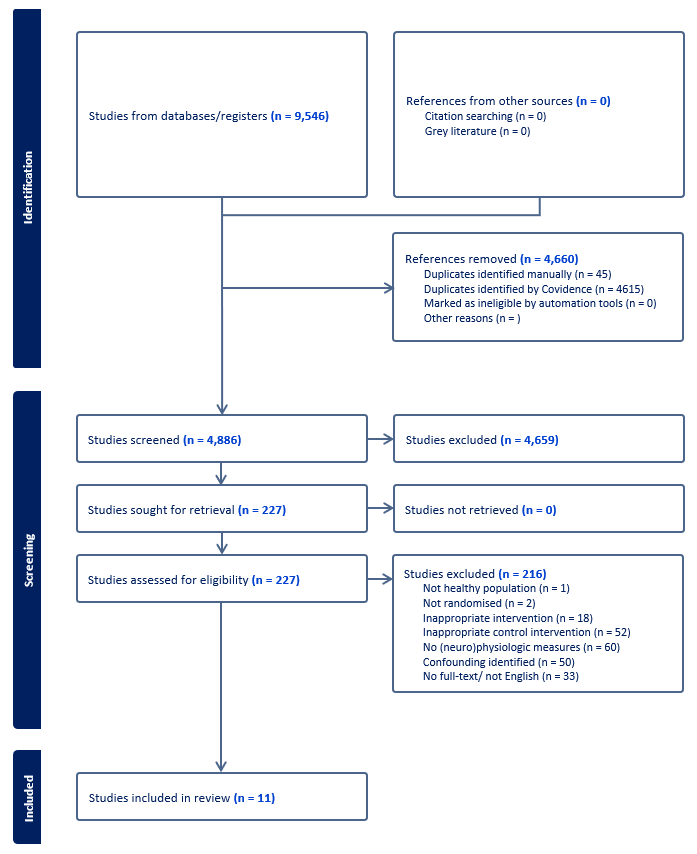


**Supplementary Figure 2**. Third literature search that combined an unrestricted (except for human participants) literature search for (“cognitive effort” OR “mental effort”) and final iteration of the initial search (with restrictions on human participants, published in English, and between the dates of Feb, 2023 to June, 2024).

**Supplementary table 1.** Demographic information and outcome measures of (neuro)physiologic function

| Citation | Participants (number and sex) | Age (years) | Experimental design | Experimental intervention | | Control intervention | | Outcome(s) |
| --- | --- | --- | --- | --- | --- | --- | --- | --- |
|  |  |  |  | Type | Duration | Type | Duration |  |
| Angius, 2022 | 17 (17M) | 26 ± 2 | Within | Stroop (mixed) –  word on red; colour on remaining | 30 min | Video | 30 min | **Neuroimaging**  Left PFC oxygenated, deoxygenated, and total hemoglobin (%) |
| Batista, 2021 | 15 (15M) | 30 ± 8 | Within | Stroop (mixed) – word on red; colour on remaining | 30 min | Video (10 min) + Relaxation (20 min) | 30 min | **Physiologic**  Mean heart rate (bpm) |
| Behrens, 2018a – Young adults | 16 (8F, 8M) | 24.9 ± 1.4 | Within | Inhibition task – Stop-signal test | 90 min | Video | 90 min | **Physiologic**  LF (ms^2^) and RMSSD (ms) HRV |
| Behrens, 2018b – Older adults | 16 (10F, 6 M) | 72.2 ± 4.4 | Within | Inhibition task – Stop-signal test | 90 min | Video | 90 min | **Physiologic**  LF (ms^2^) and RMSSD (ms) HRV |
| Boat, 2021 | 13 (13M) | 20 ± 1 | Within | Stroop (mixed) – word on red; colour on remaining | 4 min | Stroop (word) – 100% congruent) | 4 min | **Physiologic**  Plasma cortisol |
| Brown, 2019A | 25 (13M, 12F) | 20.16 ± 1.48 | Within | AX-CPT | 50 min | Video | 50 min | **Physiologic**  Mean heart rate (bpm) |
| Brown, 2019B | 36 (20F, 16M) | 19.44 ± 1.42 | Within | Stroop (colour) - 100% incongruent | 10 / 135 trials | Video | 10 min | **Physiologic**  Mean heart rate (bpm) |
| Brownsberger, 2013 | 12 (8M, 4F) | 24 ± 5 | Within | AX-CPT | 90 min | Video | 90 min | **Physiologic**  Mean heart rate (bpm)  **Neuroimaging**  Frontal cortex alpha and beta EEG (logμV^2^) |
| Budini, 2022 | 29 (19M, 10F) | 25.1 ± 5 | Between | Switching task | 100 min | Video | 100 min | **Physiologic**  Capillary oxygen saturation (%)  Mean heart rate (bpm)  Systolic and diastolic blood pressure (mmHg) |
| Campos, 2019 | 13 (9M, 4F) | 19.5 ± 3 | Within | Stroop (mixed) – word on red; colour on remaining | 30 min | Video | 30 min | **Physiologic**  HF and LF (ms^2^) HF:LF ratio, RMSSD (ms) HRV  Salivary cortisol (ng/mL)  Blood glucose (mg/dL)  Blood lactate (mmol/L) |
| Clark, 2019a – Competitively trained males | 10 (10M) | 27.4 ± 6.3 | Within | Mixed - Stroop (colour) and N-back task | 30 min | Video | 30 min | **Physiologic**  Mean heart rate (bpm)  Plasma lactate, glucose, sodium, potassium (mM)  Plasma cortisol (μg/dL)  **Neuroimaging**  Left PFC oxygenated, deoxygenated, and total hemoglobin (a.u.) |
| Clark, 2019b – Untrained healthy males | 10 (10M) | 25.8 ± 4.6 | Within | Mixed - Stroop (colour) and N-back task | 30 min | Video | 30 min | **Physiologic**  Mean heart rate (bpm)  Plasma lactate, glucose, sodium, potassium (mM)  Plasma cortisol (μg/dL)  **Neuroimaging**  Left PFC oxygenated, deoxygenated, and total hemoglobin (a.u.) |
| Dallaway, 2022a – 2-back test MF group | 90 (52F, 38M) | 19.4 ± 1.3 | Between | 2-back test | 40 min | Video | 40 min | **Physiologic**  Mean heart rate (bpm)  Mean RMSSD and SDNN HRV (ms) |
| Dallaway, 2022b – Stroop task MF group | 90 (52F, 38M) | 19.4 ± 1.3 | Between | Stroop (colour) – 100% incongruent | 40 min | Video | 40 min | **Physiologic**  Mean heart rate (bpm)  Mean RMSSD and SDNN HRV (ms) |
| Dang, 2016 | 84 (42M, 42F) | NR | Between | Stroop (colour) – 50% incongruent | NR / 144 trials | Stroop (word) – 100% congruent) | NR / 144 trials | **Visual responses**  Eye blink ratio |
| De Lima-Junior, 2023 | 18 (18M) | 22.1 ± 2.0 | Within | Stroop (colour) – 100% incongruent | 30 min | Video | 30 min | **Visual responses**  Pupil diameter (mm) |
| Englert, 2019 | 112 (87F, 25M) | 24.77 ± 10.12 | Between | Transcription task – omit E and N | 6 min | Transcription task – no omissions | 6 min | **Visual responses**  Gaze fixations (%) |
| Fairclough, 2004 | 30 (21F, 9M) | 24.77 ± NR | Within | Stroop (colour) – 100% incongruent | 45 min | Stroop (word) – 100% congruent) | 45 min | **Physiologic**  Blood glucose (% in mmol)  Mean heart rate (bpm) |
| Ferris, 2021 | 8 (8M) | 24.3 ± 0.4 | Within | AX-CPT | 60 min | Video | 60 min | **Physiologic**  Mean heart rate (bpm) |
| Filipas, 2019 | 10 (10M) | 20.0 ± 1.2 | Within | Stroop (mixed) – word on red; colour on remaining | 30 min | Video | 30 min | **Physiologic**  Mean heart rate (bpm)  Mean RMSSD HRV (ms)  Blood lactate (mmol/L) |
| Filipas, 2018a – Stroop task MF condition | 18 (11M, 7F) | 11 ± 1.06 | Within | Stroop (colour) - 100% incongruent | 60 min | Painting Mandala | 60 min | **Physiologic**  Mean heart rate (bpm) |
| Filipas, 2018b – Arithmetic MF condition | 18 (11M, 7F) | 11 ± 1.06 | Within | Arithmetic | 60 min | Painting Mandala | 60 min | **Physiologic**  Mean heart rate (bpm) |
| Galy, 2015 | 31 (15F, 16M) | 23.3 ± 1.86 | Within | Arithmetic – Serial 36 + time pressure | 4 min | Arithmetic – Serial 5 | 4 min | **Physiologic**  Differential heart rate (bpm) |
| Gantois, 2020a – 30 min MF condition | 20 (20M) | 22.6 ± 3.3 | Within | Stroop (word) – congruency frequency NR | 30 min | Video | 30 min | **Physiologic**  Mean RMSSD, SDNN, pNN50 HRV (units NR) |
| Gantois, 2020b – 15 min MF condition | 20 (20M) | 22.6 ± 3.3 | Within | Stroop (word) – congruency frequency NR | 15 min | Video | 30 min | **Physiologic**  Mean RMSSD, SDNN, pNN50 HRV (units NR) |
| Gieseler, 2021 | 179 (128F, 51M) | 22.79 ± 3.27 | Between | Arithmetic – Serial 13 | 8 min | Arithmetic – Serial 2 | 8 min | **Physiologic**  Pre-ejection period (mmHg from baseline)  SBP (mmHg from baseline |
| Goudini, 2024 | 17 (14M, 3F) | M: 28.6 ± 4.9  F: 24.0 ± 2.6 | Within | Stroop (colour) – 50% incongruent | 30 min | Video | 30 min | **Physiologic**  Mean heart rate (bpm) |
| Habay, 2021 | 11 (4F, 7M) | 24 ± 2 | Within | Stroop (mixed) –  word on red; colour on remaining  + Individualised time pressure | 60 min | Video | 60 min | **Physiologic**  Mean heart rate (bpm)  **Neuroimaging**  EEG theta, upper and lower alpha bands, and event related potentials (N1, P2, N2, and P3b) |
| Hakim, 2022 | 12 (5F, 7M) | 28 ± 7.5  Range 25-30 | Within | Stroop (type NR) – Congruency frequency NR | 60 min | Video | 60 min | **Physiologic**  Blood glucose (% from baseline) |
| Head, 2017 | 20 (20M) | NR | Within | Sustained attention to response task (Go/no-go like assessment) | 49 min | Video | 49 min | **Physiologic**  SDNN HRV (units NR) |
| Head, 2016 | 18 (11M, 7F) | 28 ± 3.8  Range: 24-37 | Within | Vigilance task (Go/no-go like assessment) | 52 min | Video | 52 min | **Physiologic**  Mean heart rate (bpm)  Oxygen consumption (ml/kg/min) |
| Hess, 2012a – Young adults | 52 (26F, 26M) | 31.6 ± NR | Between | Arithmetic - Serial 3 | 5 min | Arithmetic – Serial 1 | 5 min | **Physiologic**  SBP and DBP (mmHg from baseline)  Mean heart rate (bpm) |
| Hess, 2012b – Older adults | 51 (24F, 27M) | 70.9 ± NR | Between | Arithmetic - Serial 3 | 5 min | Arithmetic – Serial 1 | 5 min | **Physiologic**  SBP and DBP (mmHg from baseline)  Mean heart rate (bpm) |
| Holgado, 2023 | 22 (18M, 4F) | 26.4 ± 4.46 | Within | TloadDback (individualised) | 30 min | 0-back test | 30 min | **Neuroimaging**  First dorsal interosseous muscle motor evoked potential (mV)  Left PFC oxygenated, deoxygenated, and total hemoglobin (%) |
| Ishii, 2013 | 9 (9M) | 36.8 ± 11.8 | Within | 2-back test | 75 min | 0-back test | 75 min | **Physiologic**  HF and LF (%), and HF:LF ratio HRV  **Neuroimaging**  MEG Peak voxel |
| Jacquet, 2024 | 14 (7F, 7M) | 22.6 ± 2.3 | Within | Visual attention task | 62 min | Video | 62 min | **Neuroimaging**  EEG delta, theta, alpha, beta, and gamma bands across the frontal, central, and parietal regions, and event related potentials (P1 and N1)  EEG theta, upper and lower alpha bands, and event related potentials (N1, P2, N2, and P3b) |
| Klaassen, 2013a – responders | 15 (15M) | 43.3 ± 13.4  Range: 27-61 | Within | Mixed: N-back tests, Stroop task (type NR), mental arithmetic, puzzles | 90 min | Video or magazine | 90 min | **Physiologic**  Salivary cortisol (nmol/L)  **Neuroimaging**  MRI during post-intervention working memory tasks. |
| Klaassen, 2013b – non-responders | 12 (12M) | 45.8 ± 12.6  Range: 25-60 | Within | Mixed: N-back tests, Stroop task (type NR), mental arithmetic, puzzles | 90 min | Video or magazine | 90 min | **Physiologic**  Salivary cortisol (nmol/L)  **Neuroimaging**  MRI during post-intervention working memory task. |
| Klaassen, 2014a – Young | 14 (14M) | Range: 25-35 | Within | Mixed: N-back tests, Stroop task (type NR), mental arithmetic, puzzles | 90 min | Video or magazine | 90 min | **Neuroimaging**  MRI during post-intervention working memory task. |
| Klaassen, 2014b – Middle-aged | 18 (18M) | Range: 60-61 | Within | Mixed: N-back tests, Stroop task (type NR), mental arithmetic, puzzles | 90 min | Video or magazine | 90 min | **Neuroimaging**  MRI during post-intervention working memory task. |
| Klaassen 2016b – Young | 14 (14M) | Range: 25-35 | Within | Mixed: N-back tests, Stroop task (type NR), mental arithmetic, puzzles | 90 min | Video or magazine | 90 min | **Neuroimaging**  MRI during post-intervention working memory task. |
| Klaassen 2016b – Middle-aged | 18 (18M) | Range: 50-61 | Within | Mixed: N-back tests, Stroop task (type NR), mental arithmetic, puzzles | 90 min | Video or magazine | 90 min | **Neuroimaging**  MRI during post-intervention working memory task. |
| Kosack, 2020 | 19 (19M) | 20 ± 2.8 | Within | Stroop (mixed) –  word on red; colour on remaining | 60 min | Video | 60 min | **Physiologic**  Blood lactate (mmol/L) |
| Kowalski, 2022a – Males | 15 (15M) | 20 ± 2.8 | Within | Psychomotor vigilance task | 30 min | Video | 30 min | **Neuroimaging**  Tibulus anterior motor evoked potential amplitude (mV) and cortical silent period (ms) |
| Kowalski, 2022b – Females | 15 (15F) | 24.9 ± 2.3 | Within | Psychomotor vigilance task | 30 min | Video | 30 min | **Neuroimaging**  Tibulus anterior motor evoked potential amplitude (mV) and cortical silent period (ms) |
| Le, 2021a – Neutral sequential task | Total n for citation:  120 (72F, 48F) | 18.39 ± 0.61 | Between | Stroop (colour) - 70% incongruent | 10 min | Video | 10 min | **Physiologic**  RMSSD (ms) |
| Le, 2021a – Positive sequential task | Total n for citation:  120 (72F, 48F) | 18.39 ± 0.61 | Between | Stroop (colour) - 70% incongruent | 10 min | Video | 10 min | **Physiologic**  RMSSD (ms) |
| Lopes, 2020a - Males | 16 (16M) | 25 ± 1 | Within | Stroop (mixed) –  word on red; colour on remaining | 45 min | Video | 45 min | **Physiologic**  Blood lactate (mmol/L) |
| Lopes, 2020b - Females | 15 (15F) | 25 ± 1 | Within | Stroop (mixed) –  word on red; colour on remaining | 45 min | Video | 45 min | **Physiologic**  Blood lactate (mmol/L) |
| MacMahon, 2014 | 20 (18M, 2F) | 25.4 ± 3.24 | Within | AX-CPT | 90 min | Video + AX-Continuous performance task (last 6 min) | 90 min | **Physiologic**  Average heart rate (bpm)  Blood lactate (units NR) |
| Marcora, 2009 | 16 (10M, 6F) | 26 ± 3 | Within | AX-CPT | 90 min | Video | 90 min | **Physiologic**  Average heart rate (bpm)  Blood glucose (units NR) |
| Martin, 2016a – Professional road cyclists | 11 (11M) | 23.4 ± 6.4 | Within | Stroop (mixed) – word on red; colour on remaining | 30 min | Screen viewing | 10 min | **Physiologic**  Blood lactate (mmol/L) |
| Martin, 2016b – Recreational road cyclists | 9 (9M) | 25.6 ± 5.3 | Within | Stroop (mixed) – word on red; colour on remaining | 30 min | Screen viewing | 10 min | **Physiologic**  Blood lactate (mmol/L) |
| Matuz, 2021 | 41 (22F, 19M) | 21.87 ± 3.18 | Between | 2-back test (gatekeeper task) | 90 min | Video | 90 min | **Physiologic**  Average heart rate (bpm)  HF and LF (ms^2^), RMSSD (ms) PNN50 (%), Poincaré cloud SD2 (units NR) HRV |
| Mlynski, 2021a – Low Evocativeness group | 57 (57NR) | 20.34 ± NR | Between | AX-CPT | 8 min | AX-CPT (easy) | 8 min | **Physiologic**  Average heart rate (bpm)  Pre-ejection period (ms), SBP, DBP, and MAP (mmHg) |
| Mlynski, 2021b – High Evocativeness group | 60 (60NR) | 20.34 ± NR | Between | AX-CPT | 8 min | AX-CPT (easy) | 8 min | **Physiologic**  Average heart rate (bpm)  Pre-ejection period (ms), SBP, DBP, and MAP (mmHg) |
| Moreira, 2018 | 32 (32M) | 15.2 ± 1.2 | Within | Stroop (mixed) –  word on red; colour on remaining | 30 min | Screen viewing (10 min) and leisure activity (20 min) | 30 min | **Physiologic**  Salivary testosterone (pmol/L), alpha-amylase (U/mL), and cortisol (nmol/L) |
| O'Keeffe, 2020a – TloadDback MF condition | 12 (7F, 5M) | 26.5 ± 3.12 | Within | TloadDback (individualised) | 16 min | TloadDback (standard) | 16 min | **Physiologic**  Average heart rate (bpm)  RMSSD (ms) and PNN50 (%) HRV  Galvanic skin conductance (µS) |
| O'Keeffe, 2020a – TloadDback MF condition | 12 (7F, 5M) | 26.5 ± 3.12 | Within | TloadDback (individualised) | 16 min | Video | 90 min | **Physiologic**  Average heart rate (bpm)  RMSSD (ms) and PNN50 (%) HRV  Galvanic skin conductance (µS) |
| O'Keeffe, 2020c – AX-CPT condition | 12 (7F, 5M) | 26.5 ± 3.12 | Within | AX-CPT | 90 min | Video | 90 min | **Physiologic**  Average heart rate (bpm)  RMSSD (ms) and PNN50 (%) HRV  Galvanic skin conductance (µS) |
| Otani, 2017 | 8 (8M) | 22 ± 0.6 | Within | Mixed: Stroop, Sternberg memory test, and RVIP | 90 min | Video | 90 min | **Physiologic**  Average heart rate (bpm)  MAP (mm/Hg) |
| Pageaux, 2013 | 10 (10M) | 22 ± 2 | Within | AX-CPT | 90 min | Video | 90 min | **Physiologic**  Average heart rate (bpm) |
| Pageaux, 2014 | 12 (8M, 4F) | 21 ± 1 | Within | Stroop (mixed) –  word on red; colour on remaining | 30 min | Stroop (word) – 100% congruent) | 30 min | **Physiologic**  Average heart rate (bpm)  Blood glucose and lactate (mmol/L) |
| Pageaux, 2015 | 12 (12M) | 25 ± 4 | Within | Stroop (mixed) –  word on red; colour on remaining | 30 min | Stroop (word) – 100% congruent) | 30 min | **Physiologic**  Average heart rate (bpm) |
| Park, 2021 | 14 (7F,7M) | 25.2 ± 3.4 | Within | Arithmetic (difficult equations) | 15 min | Arithmetic (easy equations) | 15 min | **Neuroimaging**  EEG P600 amplitude (microV) |
| Penna, 2018A | 12 (12NR) | 17.5 ± 3.63 | Within | Stroop (mixed) –  word on red; colour on remaining | 30 min | Video | 30 min | **Physiologic**  Blood lactate (mmol/L) |
| Penna, 2018B | 16 (11M, 5F) | 15.45 ± 0.51 | Within | Stroop (mixed) –  word on red; colour on remaining | 30 min | Video | 30 min | **Physiologic**  HF and LF (n.u.), HF:LF ratio, R-R interval (ms), and RMSSD (ms) HRV |
| Pires, 2018 | 8 (8M) | 29.3 ± 7.9 | Within | Rapid visual information processing test | 30 min | Mixed: video and reading | 30 min | **Neuroimaging**  PFC EEG Theta wave activity (%) |
| Proost, 2024 | 16 (7F,9M) | 21 ± 6 | Within | Stroop (mixed) –  word on red; colour on remaining + time pressure | 60 min | Video | 60 min | **Physiologic**  Blood glucose and lactate (mg/dL)  Mean heart rate (bpm)  **Neuroimaging**  EEG theta and alpha activity at frontal, parietal, and occipital regions |
| Rouse, 2013 | 77 (42F, 35M) | 19.8 ± 1.7 | Between | Stroop (mixed) –  word on blue; colour on remaining | NR | Stroop – 100% congruent) | NR | **Physiologic**  Blood glucose (mg/dL) |
| Roussey, 2018 | 11 (11M) | 27.0 ± 8.6 | Within | Stroop (mixed) –  word on red; colour on remaining | 60 min | Video | 60 min | **Physiologic**  Average heart rate (bpm)  Blood lactate (mmol/L) |
| Rozand, 2014a – Cognitive task control condition | 10 (10M) | 24.5 ± 1.4 | Within | Stroop (mixed) –  word on red; colour on remaining | 27 min | Stroop task – 100% congruent | 27 min | **Physiologic**  Average heart rate (bpm) |
| Rozand, 2014b – Video control condition | 10 (10M) | 24.5 ± 1.4 | Within | Stroop (mixed) –  word on red; colour on remaining | 27 min | Video | 27 min | **Physiologic**  Average heart rate (bpm) |
| Salihu, 2023 | 15 (4F, 11M) | 29.13 ± 7.15 | Within | Stroop (colour) – 75% incongruent | 60 min | Video | 60 min | **Neuroimaging**  Transcranial magnetic stimulation, corticospinal excitability, short and long intracortical inhibition, and intracortical facilitation |
| Schücker, 2016a – Study 1 | 12 (9F, 3M) | 29.41 ± 14.47 | Within | Stroop (mixed) –  word on red; colour on remaining | 10 min | Stroop task – 100% congruent | 10 min | **Physiologic**  Average heart rate (bpm)  Blood glucose (units NR) |
| Schücker, 2016b – Study 2 | 14 (9F, 5M) | 30.64 ± 13.11 | Within | Stroop (mixed) –  word on red; colour on remaining | 10 min | Video | 10 min | **Physiologic**  Average heart rate (bpm) |
| Shigihara, 2013 | 10 (10M) | 30.8 ± 9.4 | Within | 2-back test | 30 min | 0-back | 30 min | **Neuroimaging**  Change in MEG alpha and beta power |
| Shigihara, 2012 | 12 (12M) | 30.5 ± 9.2 | Within | 2-back test | 30 min | 0-back | 30 min | **Neuroimaging**  Change in MEG visual cortex evoked magnetic field intensity |
| Smith, 2019a – PVT MF condition | 17 (11M, 6F) | 21.4 ± 3.5 | Within | Psychomotor vigilance task | 45 min | Video | 45 min | **Physiologic**  Average heart rate  Absolute and normalised very low, LF and HF HRV (n.u.)  **Neuroimaging**  EEG delta, theta, upper alpha, lower alpha, and beta power |
| Smith, 2019b – AX-CPT MF condition | 17 (11M, 6F) | 21.4 ± 3.5 | Within | AX-CPT | 45 min | Video | 45 min | **Physiologic**  Average heart rate  Absolute and normalised very low, LF and HF HRV (n.u.)  **Neuroimaging**  EEG delta, theta, upper alpha, lower alpha, and beta power |
| Smith, 2019c – Stroop MF condition | 17 (11M, 6F) | 21.4 ± 3.5 | Within | Stroop (colour) – 50% incongruent | 45 min | Video | 45 min | **Physiologic**  Average heart rate  Absolute and normalised very low, LF and HF HRV (n.u.)  **Neuroimaging**  EEG delta, theta, upper alpha, lower alpha, and beta power |
| Smith, 2015 | 10 (10M) | 22 ± 2 | Within | AX-CPT | 90 min | Video | 90 min | **Physiologic**  Average heart rate (bpm)  Blood glucose and lactate (mmol/L) |
| Smith, 2016 | 12 (12M) | 19.3 ± 1.5 | Within | Stroop (mixed) –  word on red; colour on remaining | 30 min | Magazine reading | 30 min | **Visual responses**  Number of fixations (#/sec), fixation duration (ms), fixation percentage (%), and fixation order (#/sec) |
| Tanaka, 2012 | 18 (18M) | 30.1 ± 10.8 | Within | 2-back test | 30 min | 0-back | 30 min | **Physiologic**  HF and LF (%) and HF:LF ratio  Salivary cortisol (nmol/L)  **Neuroimaging**  EEG beta, alpha, theta, delta power |
| Timme, 2022 | 65 (43F, 22M) | 24.2 ± 3.25 | Within | Go/no-go task | ~10 min | Go/go task | ~10 min | **Visual responses**  Pupil diameter during intervention (mm) |
| Van Cutsem, 2019a – badminton players | 9 (5M, 4F) | 23 ± 3 | Within | Stroop (mixed) –  word on red; colour on remaining | 90 min | Video | 90 min | **Physiologic**  Average heart rate (bpm) |
| Van Cutsem, 2019b – healthy controls | 11 (6F, 5M) | 25 ± 4 | Within | Stroop (mixed) –  word on red; colour on remaining | 90 min | Video | 90 min | **Physiologic**  Average heart rate (bpm) |
| Van Cutsem, 2019c – combined | 20 (10F, 10M) | 25 ± 4 | Within | Stroop (mixed) –  word on red; colour on remaining | 90 min | Video | 90 min | **Physiologic**  Average heart rate (bpm)  Blood glucose (mg/dl) |
| Van Cutsem, 2022 | 20 (12F, 8M) | 23 ± 1 | Within | Stroop (mixed) –  word on red; colour on remaining + time pressure | 90 min | Video | 90 min | **Physiologic**  R-R interval, SDNN, RMSSD, pNN50, triangular index, triangular interpolation, Poincaré cloud SD1, SD2, SD1:SD2 ratio (units NR)  Breathing rate (units NR)  Average heart rate (bpm)  Blood glucose (mg/dL)  **Neuroimaging**  fMRI during pre- and post-intervention flanker tasks |
| Widyanti, 2017a – Working memory MF condition | 54 (28M, 26F) | 21.00 ± 1.35 | Within | Working memory (maintaining 3 separate counts) | NR / 80 trials | Search task | NR / 80 trials | **Physiologic**  LF HRV (units NR)  Galvanic skin response (mean score) |
| Widyanti, 2017b – Selective attention MF condition | 54 (28M, 26F) | 21.00 ± 1.35 | Within | Selective attention (maintain 1 separate counts) | NR / 80 trials | Search task | NR / 80 trials | **Physiologic**  LF HRV (units NR)  Galvanic skin response (mean score) |
| Wright, 2003a – high difficulty | 36 (36F) | NR | Between | Arithmetic: Serial 3 (subtraction) | 5 min | Arithmetic: Serial 1 (addition) | 5 min | **Physiologic**  Average heart rate (bpm)  DBP, SBP, MAP (mmHg) |
| Wright, 2003b – low difficulty post | 36 (36F) | NR | Between | Arithmetic: Serial 3 (subtraction) | 5 min | Arithmetic: Serial 1 (addition) | 5 min | **Physiologic**  Average heart rate (bpm)  DBP, SBP, MAP (mmHg) |
| Wright, 2007a – Study 1, low difficulty post task | 43 (43F) | NR | Between | Arithmetic: Serial 3 (subtraction) | 5 min | Arithmetic: Serial 1 (addition) | 5 min | **Physiologic**  DBP, SBP, MAP (mmHg) |
| Wright, 2007b – Study 1, high difficulty post task | 45 (45F) | NR | Between | Arithmetic: Serial 3 (subtraction) | 5 min | Arithmetic: Serial 1 (addition) | 5 min | **Physiologic**  DBP, SBP, MAP (mmHg) |
| Wright, 2007c – Study 2, low difficulty post task | 44 (44M) | NR | Between | Arithmetic: Serial 3 (subtraction) | 5 min | Arithmetic: Serial 1 (addition) | 5 min | **Physiologic**  DBP, SBP, MAP (mmHg) |
| Wright, 2007d – Study 2, high difficulty post task | 44 (44M) | NR | Between | Arithmetic: Serial 3 (subtraction) | 5 min | Arithmetic: Serial 1 (addition) | 5 min | **Physiologic**  DBP, SBP, MAP (mmHg) |
| Wright, 2008a – Arithmetic post task | 50 (50F) | NR | Between | Attentional task:  Rule – circle H next to vowel | 5 min | Attentional task:  Rule – circle H | 5 min | **Physiologic**  DBP, SBP, MAP (mmHg)  Average heart rate (bpm) |
| Wright, 2008b – Stroop post task | 49 (49F) | NR | Between | Attentional task:  Rule – circle H next to vowel | 5 min | Attentional task:  Rule – circle H | 5 min | **Physiologic**  DBP, SBP, MAP (mmHg)  Average heart rate (bpm) |
| Wylie, 2017 | 23 (14F, 9M) | 41.7 ± 12.4 | Within | 2-back | NR | 0-back | NR | **Neuroimaging**  fMRI of anterior cingulate cortex during interventions |

**Notes:** Upper- and lower-case letters in the study column represent different citations published in the same year and independent datasets within a study respectively. Values in red are Standard error of the mean. AX-CPT is the AX-Continuous performance task, DBP is diastolic blood pressure, EEG is electroencephalography, F is female, HRV is heart rate variability, LF is low frequency, M is male, MAP is mean arterial pressure, MEG is magnetoencephalography, MRI is magnetic resonance imaging, NR is not reported, PFC is prefrontal cortex, pNN50 is successive percentage of RR intervals, RMSSD is root mean square of successive differences, RVIP is rapid visual information processing task, SDNN is standard deviation of NN intervals, SBP is systolic blood pressure.


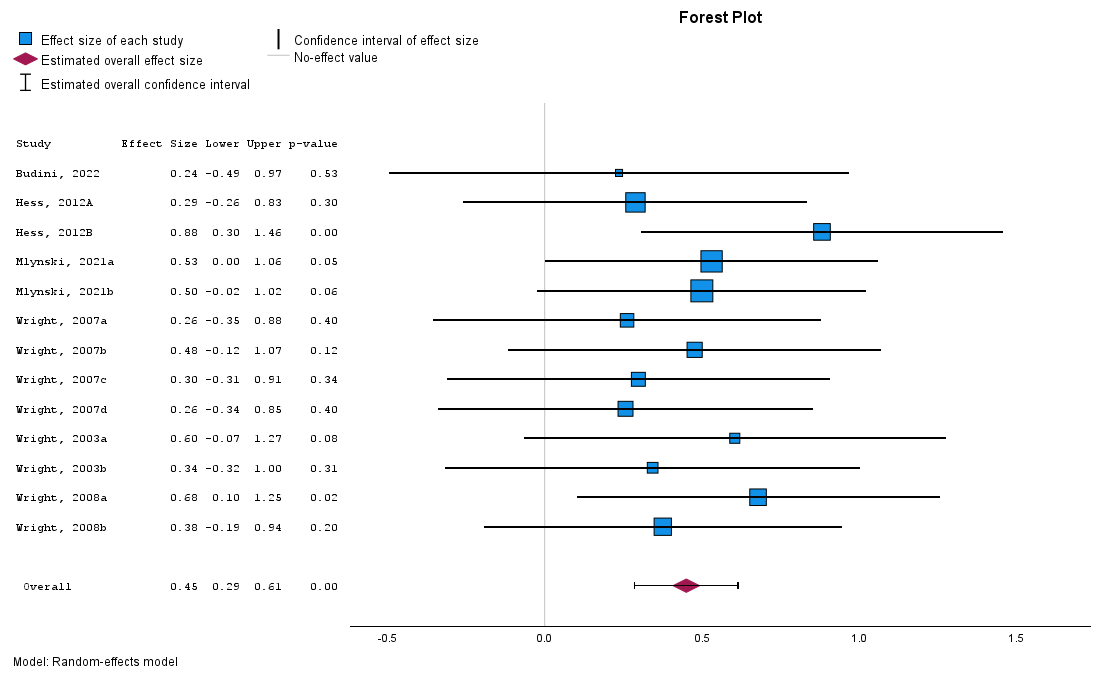


**Supplementary Figure** **2.** Overall analysis of diastolic blood pressure. The size of the blue squares is proportional to the weight of the study. The red diamond represents the overall effect. Error bars are 95% confidence intervals


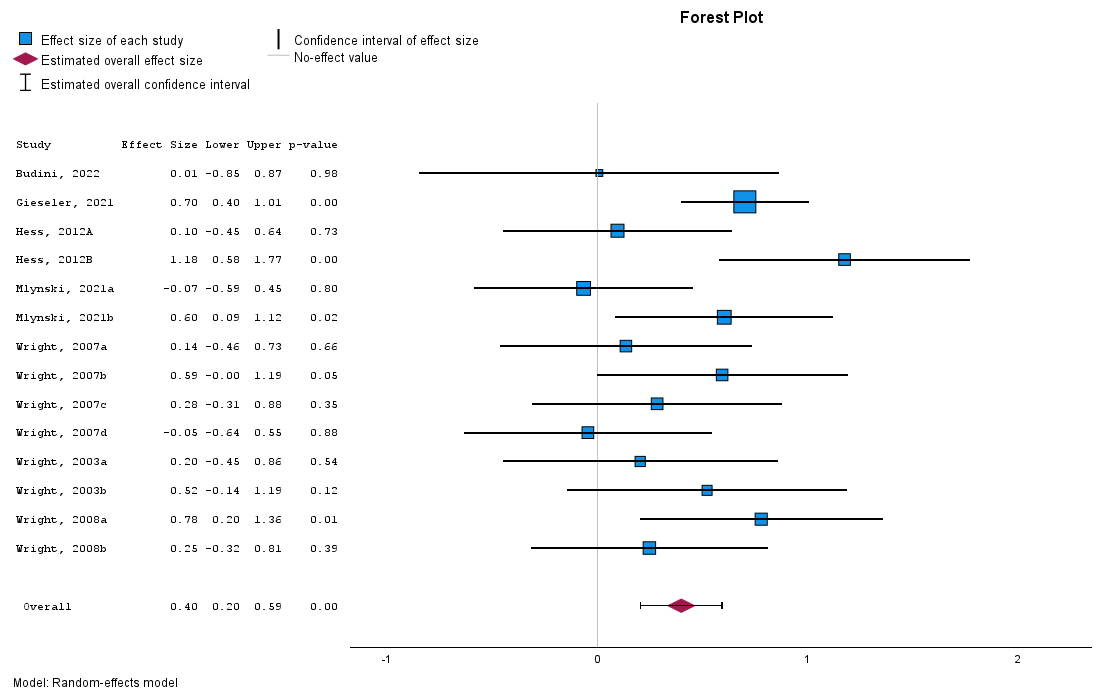


**Supplementary Figure** **3.** Overall analysis of systolic blood pressure. The size of the blue squares is proportional to the weight of the study. The red diamond represents the overall effect. Error bars are 95% confidence intervals


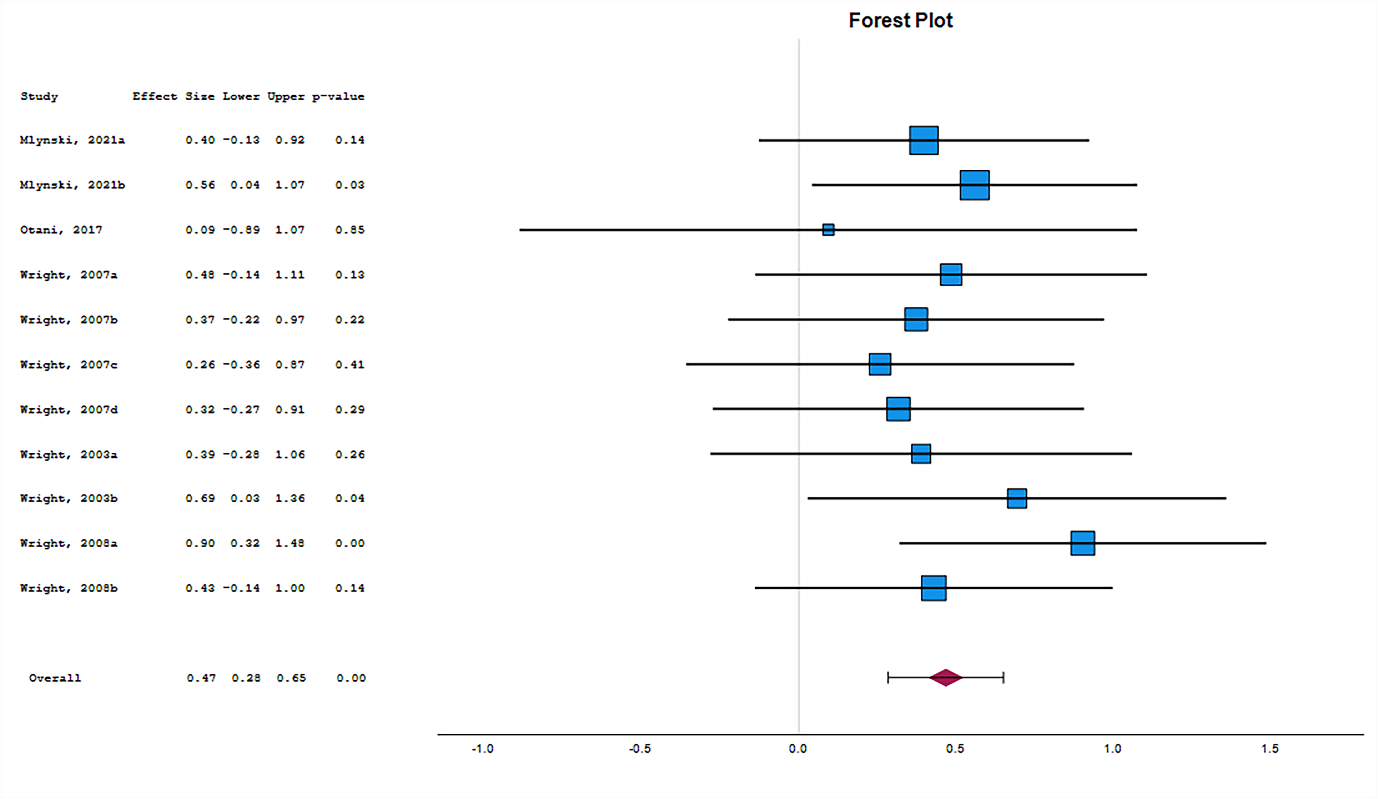


**Supplementary Figure** **4.** Overall analysis of mean arterial pressure. The size of the blue squares is proportional to the weight of the study. The red diamond represents the overall effect. Error bars are 95% confidence intervals
